# Polymorphic α‐Glucans as Structural Scaffolds in *Cryptococcus* Cell Walls for Chitin, Capsule, and Melanin: Insights From ^13^C and ^1^H Solid‐State NMR

**DOI:** 10.1002/anie.202510409

**Published:** 2025-07-15

**Authors:** Ankur Ankur, Jayasubba Reddy Yarava, Isha Gautam, Faith J. Scott, Frederic Mentink‐Vigier, Christine Chrissian, Li Xie, Dibakar Roy, Ruth E. Stark, Tamara L. Doering, Ping Wang, Tuo Wang

**Affiliations:** ^1^ Department of Chemistry Michigan State University East Lansing MI USA; ^2^ National High Magnetic Field Laboratory Florida State University Tallahassee FL USA; ^3^ Department of Chemistry and Biochemistry Florida State University Tallahassee FL USA; ^4^ Department of Chemistry and Biochemistry City College of New York New York NY USA; ^5^ CUNY Institute for Macromolecular Assemblies The City University of New York New York NY USA; ^6^ Department of Molecular Microbiology Washington University in St. Louis School of Medicine St. Louis MO USA; ^7^ Departments of Microbiology, Immunology & Parasitology Louisiana State University Health Sciences Center New Orleans LA USA

**Keywords:** Capsules, *Cryptococcus neoformans*, Fungal cell wall, Melanin, Solid‐state NMR

## Abstract

*Cryptococcus* species are major fungal pathogens responsible for life‐threatening infections in approximately a million individuals globally each year, with alarmingly high mortality rates. These fungi are distinguished by a distinctive cell wall architecture further reinforced by two virulence‐associated layers, melanin and capsule, rendering them insensitive to antifungal agents targeting the cell wall, such as echinocandins. The molecular interplay between these three biomolecular layers remains poorly understood. Here, we employ solid‐state NMR spectroscopy to examine intact cells of both wild‐type and capsule‐deficient strains of *C. neoformans*, along with melanized cells. High‐resolution ^13^C and ^1^H data revealed five distinct structural forms of α‐1,3‐glucans that play versatile roles in forming the rigid cell wall scaffold by interacting with chitin microfibrils and chitosan, and in stabilizing the mobile matrix by associating with β‐1,6‐glucan and a small fraction of β‐1,3‐glucan. Two primary forms of α‐1,3‐glucans were distributed throughout the wall, capable of hosting melanin deposition in the inner domain and capsule attachment on the cell surface. These findings offer a paradigm shift in understanding the cryptococcal cell wall and its interaction with two key virulence factors on opposite sides, raising critical biochemical questions that could inform the development of more effective antifungal treatments for cryptococcosis.

## Graphical abstract

***Cryptococcus neoformans* cell wall α‐1,3‐glucans as dual anchors for melanin and capsule**: Solid‐state NMR of intact *C. neoformans* cells reveals five forms of α‐1,3‐glucans that structurally bridge melanin and capsule layers. These polysaccharides interact with chitin, chitosan, and β‐glucans to create a molecular scaffold that underlies virulence and antifungal resistance.

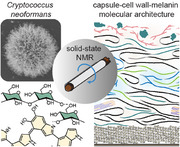

## Introduction

*Cryptococcus* species are a group of encapsulated basidiomycetous fungi causing life‐threatening infections in immunocompromised and immunocompetent individuals.^[^ ^1^ ^]^ These fungi produce characteristic virulence factors, including the antiphagocytic polysaccharide capsule, antioxidant melanin pigment, and extracellular proteases, including ureases and phospholipases.^[^ ^2^, ^3^ ^]^ Each year, approximately 200000 cases of cryptococcal meningitis are reported worldwide, with mortality rates of 20%–70% that vary significantly depending on disease severity and patient health status.^[^ ^4^, ^5^ ^]^ Untreated disease is uniformly fatal. *Cryptococcus neoformans* and *Cryptococcus gattii* are the primary species responsible for these infections, with *C. neoformans* accounting for 90% of clinical isolates.^[^ ^6^, ^7^ ^]^ Pulmonary infections are established upon inhalation of spores or desiccated yeast cells.^[^ ^8^ ^]^ In immunocompromised individuals, particularly when associated with HIV/AIDS, the infection can disseminate, enabling the pathogen to traverse the blood‐brain barrier and induce meningoencephalitis—an inflammatory condition affecting the brain and its surrounding tissues.^[^ ^9^, ^10^ ^]^

The standard treatment regimen for cryptococcosis involves amphotericin B, flucytosine, and fluconazole, typically administered for 6 to 12 months; however, this prolonged therapy is both financially burdensome and associated with significant adverse effects.^[^ ^11^, ^12^ ^]^ Recent antifungal research has focused on targeting fungal cell wall biosynthesis, leading to the successful development of echinocandins, which inhibit β‐1,3‐glucan synthesis—an essential structural component of most fungal cell walls.^[^ ^13^ ^]^ However, echinocandins exhibit limited efficacy against *Cryptococcus* species, with the underlying mechanisms for their reduced activity remaining poorly understood but likely attributed to the unique cell wall architecture of *Cryptococcus* species.^[^ ^14^, ^15^ ^]^

The cryptococcal cell wall is a dynamic, multilayered structure essential for virulence, immune evasion, and stress resistance.^[^ ^8^, ^16^ ^]^ Decades of chemical and imaging analyses have led to the proposition of a two‐layered model of cryptococcal cell walls, in which the inner layer comprises an alkali‐insoluble meshwork of β‐glucans and chitin/chitosan, and the outer layer consists of an alkali‐soluble fraction containing α‐ and β‐glucans.^[^ ^16^, ^17^, ^18^ ^]^ Unlike in other yeasts, where β‐1,3‐glucan is predominant, β‐glucans in the *Cryptococcus* cell wall are primarily β‐1,6‐linked, forming covalent linkages with β‐1,3‐glucan, chitin, and cell wall proteins, while β‐1,3‐glucan is less abundant.^[^ ^15^, ^19^ ^]^ The cryptococcal cell wall is further associated with two additional layers of biomacromolecules that both serve as key virulence factors: a capsular layer coating the surface and melanin deposited between the cell wall polysaccharides and the plasma membrane.^[^ ^20^, ^21^, ^22^, ^23^ ^]^

The cryptococcal capsule, which mediates host–pathogen interactions, is primarily composed of glucuronoxylomannan (GXM), with minor fractions of glucuronoxylomannogalactan (GXMGal) and mannoproteins.^[^ ^24^, ^25^, ^26^ ^]^ These capsular polysaccharides are found both attached to the cell wall and released as exopolysaccharides.^[^ ^27^ ^]^ Under nutrient‐deficient conditions, *Cryptococcus* polymerizes external polyphenolic compounds, leading to melanin deposition in its cell wall.^[^ ^28^, ^29^ ^]^ These melanin granules form layered structures that protect fungi against oxidative stress, contribute to virulence, and allow small molecules, such as sugars and amino acids, to pass while restricting access by larger antifungal compounds.^[^ ^30^, ^31^ ^]^ Current knowledge of capsular carbohydrates is based mainly on exopolysaccharides isolated from culture supernatants, rather than those associated with the cell wall, while studies on melanin have focused on extracts called melanin ghosts, making it challenging to understand the interaction patterns of these complex and heterogeneous polymers within the cell wall.^[^ ^27^, ^32^ ^]^

Recently, solid‐state NMR spectroscopy has been introduced to enable high‐resolution structural analysis of polysaccharides within intact fungal cells, thereby eliminating the need for cell disruption or fractionation and allowing the investigation of native physiological architectures and interactions without perturbation. Here, we use ^13^C and ^1^H solid‐state NMR, enhanced by the sensitivity‐boosting Dynamic Nuclear Polarization (DNP) technique,^[^ ^33^, ^34^, ^35^, ^36^ ^]^ to explore the structural polymorphism and assembly of carbohydrate polymers in intact cells of wild‐type and acapsular *C. neoformans* strains. Our detailed examination of the cell wall reveals that β‐1,6‐glucan is the predominant component of the mobile matrix, accompanied by smaller amounts of β‐1,3‐glucans and mannoproteins, and is also present—albeit less abundantly—in the rigid phase alongside chitin, chitosan, and α‐1,3‐glucans. α‐1,3‐glucans dominate the rigid core and exhibit five distinct structural forms, which variously contribute to mechanical reinforcement, mobile matrix integration, capsular polysaccharide anchoring, and melanin deposition. These novel structural insights elucidate the organization of *C. neoformans* cell walls, provide a molecular perspective on their interface with melanin and the capsule, and underscore the critical and diverse structural roles of α‐glucans, highlighting their potential as novel therapeutic targets.

## Results

### Predominance of α‐1,3‐Glucans in the Rigid Core of Cell Wall in *Cryptococcus*

**Figure 1:**
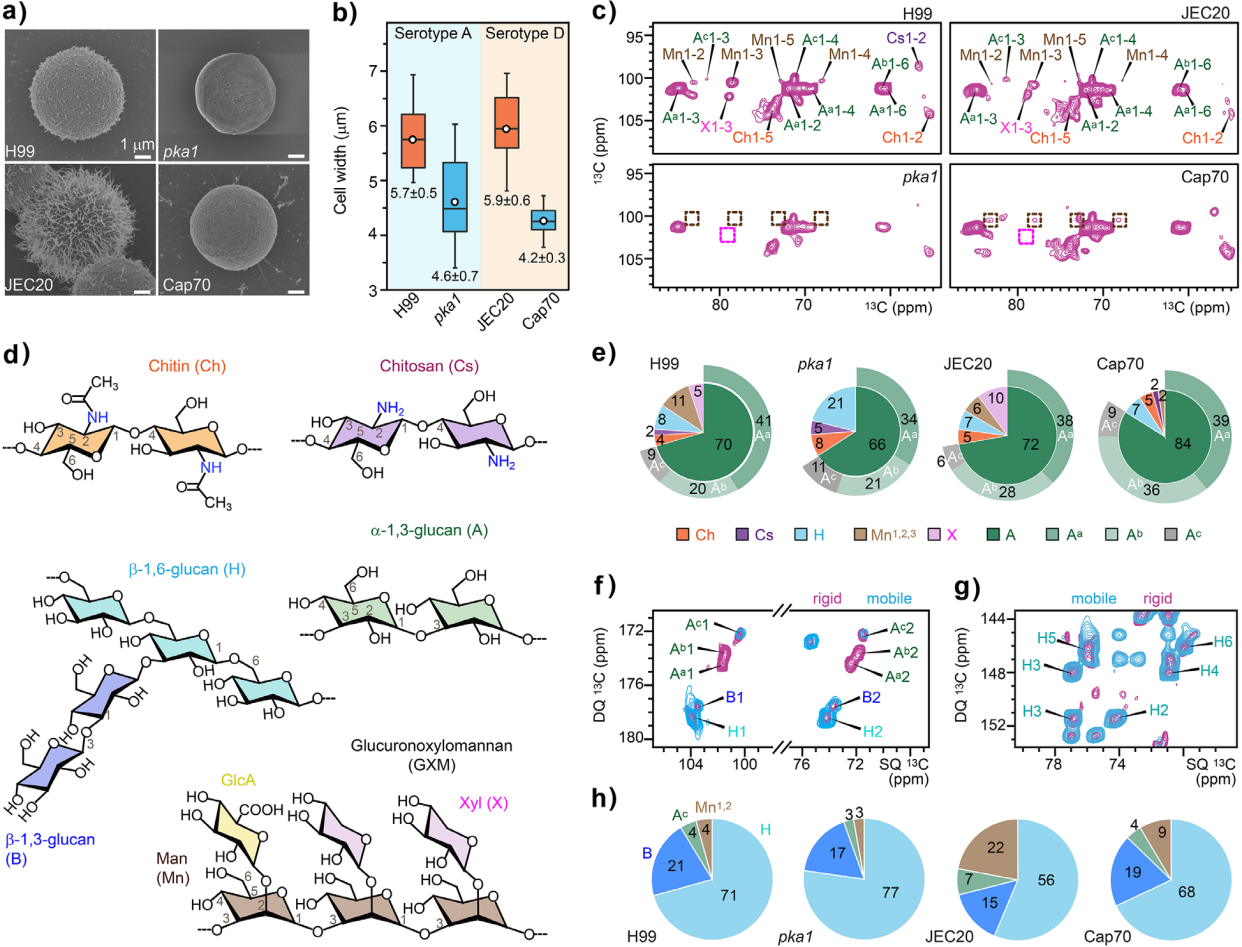
Polysaccharide composition varies in wildtype strains and capsule mutants of *C. neoformans*. a) SEM images of *C. neoformans* serotype A strains (wildtype H99 and acapsular *pka1*) and serotype D (wildtype JEC20 and hypocapsular Cap70). b) Total cell diameter measured from SEM images. Boxes represent the interquartile range (IQR), with whiskers extending to 1.5 times the IQR. Open circles: average; horizontal lines: median. Sample sizes: H99 and *pka1* (*n* = 24), JEC20 (*n* = 19), and Cap70 (*n* = 32). Standard deviations are reported as errors. Statistical analysis was performed using a t‐test with one‐tailed comparison between serotype A strains H99 and *pka1*, and serotype D strains JEC20 and Cap70. Statistically significant: *p‐value ≤0.05. c) 2D ^13^C‐^13^C CORD correlation spectra of four *C. neoformans* samples. The absence of α‐1,2,3‐mannose and xylose signals in the acapsular *pka1* mutant is highlighted with dashed brown and magenta squares, respectively, with intensity reductions observed in the hypocapsular Cap70 mutant. Observed carbohydrates include α‐1,3‐glucan (A), chitin (Ch), chitosan (Cs), β‐1,6‐glucan (H), α‐1,2,3‐mannose (Mn), and xylose (X). Three subtypes of α‐1,3‐glucan (A^a^, A^b^, and A^c^) are indicated by superscripts. d) Simplified structural representations of key polysaccharides, with NMR abbreviations and key carbon sites labeled. e) Molar composition of rigid polysaccharides estimated from resolved cross‐peak volumes in 2D ^13^C CORD spectra. f) and g) Overlay of refocused J‐INADEQUATE spectra obtained via direct polarization (DP) for mobile molecules (cyan) and cross polarization (CP) for rigid molecules (purple) reveals (f) β‐1,6‐glucan dominance in the mobile fraction and (g) preferential localization of A^a^ and A^b^ in the rigid fraction, while A^c^ is distributed across both rigid and mobile phases. h) Molar composition of mobile polysaccharides estimated from well‐resolved peaks in DP refocused J‐INADEQUATE spectra.

*Cryptococcus* species were initially classified into subtypes based on capsular polysaccharide antigenicity, but are now distinguished by DNA sequencing, ecological characteristics, and pathobiological evidence.^[^ ^37^, ^38^, ^39^, ^40^ ^]^ In this study, we selected several representative strains of the dominant pathogen *C. neoformans* for examination. To enable studies of the capsule, we compared a clinical wild‐type strain H99 with an acapsular *pka1* mutant, both of serotype A and in the same background, as well as an environmental strain JEC20 with a hypocapsular Cap70 mutant, both of serotype D (also termed *C. deneoformans*) and in the same background.^[^ ^41^, ^42^ ^]^ These mutants either completely lack or significantly suppress capsule formation on the cell surface, as confirmed by SEM imaging (Figure 1a). The mean of total cell diameter decreased from 5.7 µm in H99 cells to 4.6 µm in *pka1* cells and from 5.9 µm in JEC20 cells to 4.2 µm in Cap70 cells (Figure 1b and Table S1).

Rigid polysaccharides were analyzed using a 2D ^13^C‐^13^C CORD correlation experiment,^[^ ^43^ ^]^ revealing strong signals for α‐1,3‐glucans, along with weaker signals for chitin, chitosan, and β‐1,6‐glucan (Figures 1c,d; S1 and Table S2). Intensity analysis indicated that α‐1,3‐glucans constitute 66%–84% of the rigid polysaccharides across the four strains, while chitin and chitosan together account for 6%–13% (Figure 1e and Table S3). These findings suggest that α‐1,3‐glucan plays a critical and unanticipated role in maintaining the mechanical scaffold of the cell wall, likely by associating with the microfibrils formed by chitin and chitosan.

Unexpectedly, signals corresponding to capsular components, including the α‐1,2,3‐linked mannose residues of the glucuronoxylomannan backbone and its xylose branches, were also detected within the rigid polysaccharides of H99 and JEC20 cells (Figures 1c and S2). These signals were entirely absent in the acapsular *pka1* mutant. In the hypocapsular Cap70 mutant, xylose signals were depleted, while α‐1,2,3‐linked mannose residues were retained but at reduced intensity. Previous studies have identified capsule molecules in the mobile phase of *C. neoformans* cells cultured in capsule‐inducing media,^[^ ^44^ ^]^ and our observations show that capsular polysaccharides can be structurally rigidified through interactions with cell wall polysaccharides.

### Identification of Three Structurally and Dynamically Distinct Forms of α‐1,3‐Glucans

Three magnetically distinct subtypes of α‐1,3‐glucans—designated as types a, b, and c—were unequivocally identified within the rigid fraction, exhibiting a sequentially decreasing prevalence within each sample (Figures 1c and S3). Type‐a represents the most prevalent allomorphic form, comprising 34%–41% of the rigid carbohydrate fraction across all samples (Figure 1e). Type‐b accounts for 20%–36% of the rigid fraction, while type‐c extends from the rigid phase into the mobile phase, constituting 6%–11% of the rigid fraction (Figure 1e) and 3%–7% of the mobile fraction, as demonstrated later. In Cap70 cells, type‐b α‐1,3‐glucan was remarkably enriched, reaching levels comparable to type‐a in this mutant, suggesting a possible compensatory adaptation to the hypocapsular state.

The three spectroscopically distinguishable forms of α‐1,3‐glucans arise from local structural perturbations, including conformational variations and differential molecular interactions with neighboring components. Type‐a and type‐b exhibited identical ^13^C chemical shifts at C1 and C3 (101.4–101.6 and 85.0–85.5 ppm, respectively), the carbon sites involved in glycosidic linkages between adjacent sugar units along the polymer chain, indicating that they share the same helical screw conformation. However, these two forms are best distinguished by their C4, C5, and C6 chemical shifts, which reflect differences in hydroxymethyl conformation related to the exocyclic ─CH_2_OH group. In contrast, type‐c exhibited significantly lower ^13^C chemical shifts at C1 (100.4 ppm) and C3 (81.9 ppm), indicating an entirely restructured helical screw conformation compared to the other forms.

These α‐1,3‐glucan allomorphic forms also have distinct dynamic properties: while all three were identified within the rigid fraction, a small amount of type‐c was also detected in the mobile fraction (Figures 1f; S3 and S4). This specific form of α‐glucan may serve as a transitional component between the rigid α‐glucan and chitin domains and the dynamic matrix primarily composed of β‐1,6‐glucan (56%–77% of the mobile fraction; Figure 1g,h), β‐1,3‐glucan (15–21%), and some α‐1,2‐linked mannose residues, likely derived from mannoproteins (Figure 1h and Table S4). This finding provides evidence for the critical role of α‐1,3‐glucan in mediating physical interactions that bridge rigid and dynamic domains.

In the mobile phase, we observed abundant β‐1,6‐glucan, which is expected as it is unusually rich in *Cryptococcus* compared to other yeasts such as *Saccharomyces cerevisiae*.^[^ ^15^, ^19^ ^]^ The structure of cryptococcal β‐1,6‐glucan consists of short chains, often branched with β‐1,3‐glucan, and our data further identified it as the dominant component of the mobile matrix. The mobile phase also contains proteins and lipids; however, their widespread distribution throughout the cell precluded a focused analysis of their specific contributions to cell wall structure (Figure S5).

### Rigid α‐glucan Forms Interact with Capsules to Form Dehydrated Domains on Cell Surface

**Figure 2:**
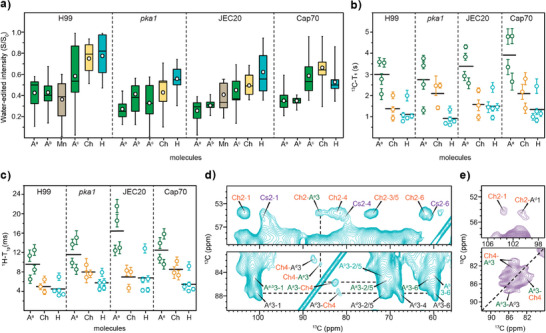
Hydration, dynamics, and DNP spectra reveal α‐glucan interactions with capsule and chitin. a) Intensity ratios (*S*/*S*_0_) between water‐edited peak spectra (*S*) and control spectra (*S*_0_) showing the extent of water association for α‐1,3‐glucan (A), chitin (Ch), 1,2,3‐linked mannan (Mn), and β‐1,6‐glucan (H), with the superscripts indicating the respective subtypes. Open circles: average; horizontal lines: median. Data size: A^a^ (*n* = 16) in all four samples; A^b^, A^c^, Ch, H (*n* = 9 each) in all; Mn (*n* = 9) in H99 and JEC20 only. b) ^13^C‐T_1_ relaxation time constants of rigid cell wall polysaccharides. c) ^1^H‐T_1ρ_ relaxation time constants of rigid polysaccharides. For both panels b and c, error bars are s.d. of the fit parameters and horizontal lines represent the average. d) DNP‐enhanced 2D ^13^C‐^13^C DARR spectrum of H99 cells. A new, minor form of α‐1,3‐glucan (A^d^) is resolved. Intermolecular cross peaks were observed between chitin and type‐a and type‐d α‐1,3‐glucans, with dashed lines to mark the intramolecular and intermolecular cross peaks of these two α‐1,3‐glucan subtypes. e) DNP‐enhanced 2D ^13^C‐^13^C PAR spectrum with 15 ms recoupling time. Top: observation of a cross peak between chitin and A^d^. Bottom: the dashed line indicates the diagonal of the C3 sites, with off‐diagonal intensities showing intermolecular cross peaks.

Hydration profiles of rigid biopolymers were analyzed using water ^1^H polarization transfer to carbohydrates via water‐editing experiments, where the *S*/*S*_0_ intensity ratios between water‐edited (*S*) and control (*S*_0_) spectra reflect the extent of water retention at specific carbon sites (Figures 2a and S6).^[^ ^45^, ^46^, ^47^ ^]^ Two α‐1,3‐glucan subtypes, a and b, were the least hydrated molecules in *Cryptococcus* cell walls, with average *S*/*S*_0_ values of 0.26–0.43 across all four strains (Figure 2a and Table S5). Meanwhile, in the examination of the molecular motions of rigid polysaccharides through NMR relaxation measurements, the resolvable carbon sites of type‐a α‐1,3‐glucan exhibited the longest ^13^C‐T_1_ and ^1^H‐T_1ρ_ relaxation time constants across the four strains, with average values of 2.7–3.9 s (Figures 2b; S7 and Table S6) and 9.7–12.6 ms (Figures 2c and S8), respectively. These values are significantly higher than those of the chitin and β‐1,6‐glucans in the rigid phase, indicating that α‐1,3‐glucans experience highly restricted molecular motion on both nanosecond and microsecond timescales. The combination of restricted dynamics and limited hydration suggests that types‐a and b α‐1,3‐glucans aggregate into large complexes that effectively exclude bulk water. In contrast, type‐c α‐1,3‐glucan was not only more hydrated but also more heterogeneously hydrated compared to other subforms (Figure 2a). This further supports the notion that type‐c α‐1,3‐glucan plays a crucial role in bridging the rigid and mobile phases of the α‐1,3‐glucan matrix (Figure 1e,h).

β‐1,6‐glucans are the best‐hydrated rigid polysaccharide in *C. neoformans*, with high *S*/*S*_0_ averages of 0.53–0.77 (Figure 2a). β‐1,6‐glucan also exhibited short ^13^C‐T_1_ (0.9–1.5 s) and ^1^H‐T_1ρ_ (4.4–6.8 ms) relaxation times— about half to two‐thirds those of type‐a α‐1,3‐glucans (Figure 2b,c). β‐1,6‐glucan is the most dynamic molecule, maintaining substantial hydration even within the rigid core. Chitin displays intermediate rigidity and hydration profiles, typically between β‐1,6‐glucan and type‐a α‐1,3‐glucan. Chitin forms a microfibrillar structure primarily through antiparallel chain packing but is not the most rigid molecule in the cell wall. This may be due to a high level of deacetylation to chitosan, estimated to be 30%–40% based on molar composition (Figure 1e), which confers dynamics on these microfibrils.

Unexpectedly, 1,3‐linked mannose residues, which compose the backbone of GXM, exhibited low *S*/*S*_0_ values, averaging 0.38 and 0.41 in H99 and JEC20 cells, respectively. This indicates significant dehydration of capsular polysaccharides in both wild‐type serotypes A and D strains (Figure 2a), which is counterintuitive because capsular GXM is surface‐exposed and would therefore be expected to be well‐hydrated. A plausible explanation is that GXM associates with α‐1,3‐glucan subtypes a and b, which not only rigidifies GXM but also forms a dehydrated layer on the cell surface. This structural organization contrasts with cryptococcal capsules produced in capsule‐inducing media, where GXM is loosely associated with cell wall molecules, thus remaining mobile and solubilized.^[^ ^44^ ^]^

Interestingly, the two serotypes responded differently to the absence or reduction of capsular polysaccharides. In serotype A, water association decreased substantially in the *pka1* mutant compared to wild‐type H99 cells (Figure 2a), with average *S*/*S*_0_ ratios dropping from 0.42 to 0.27 for type‐a α‐glucan, 0.58 to 0.33 for type‐c α‐glucan, 0.77 to 0.56 for β‐1,6‐glucan, and 0.75 to 0.43 for chitin. In contrast, serotype D exhibited increased hydration for most cell wall polysaccharides, with *S*/*S*_0_ values rising from 0.26 in JEC20 to 0.37 in Cap70 cells for type‐a α‐glucan, 0.45 to 0.59 for type‐c α‐glucan, and 0.49 to 0.67 for chitin, except for β‐1,6‐glucan, whose hydration level decreased. Therefore, different serotypes and mutants may exhibit variations in cell wall organization. However, a consistent observation is that chitin dynamics became more confined in both acapsular and hypocapsular mutants (Figure 2b,c). This suggests strengthened interactions with α‐glucans, likely due to increased availability of interaction sites on types‐a and b α‐glucans after GXM removal.

### Two Specific α‐Glucans Interact with Chitin to Form Partially Ordered Domains in the Cell Wall

We used DNP to enhance the NMR sensitivity for polysaccharides in H99 cells by 11‐fold through polarization transfer from electrons in the biradical AsymPol‐POK to the ^1^H and then ^13^C nuclei in these cellular biomolecules.^[^ ^33^, ^48^ ^]^ The observed signals arose from partially ordered molecules, including chitin, chitosan, types‐a and b α‐glucans, and a newly identified minor form, type‐d α‐1,3‐glucan (labeled as A^d^ in Figure 2d). This new form of α‐1,3‐glucan exhibited unique C3 and C4 chemical shifts at 87.6 and 67.8 ppm, with weak signals detectable only through DNP enhancement. Meanwhile, signals from mobile and semi‐mobile molecules, such as β‐1,6‐glucan and type‐c α‐1,3‐glucan, were broadened out due to a diverse ensemble of conformations trapped at the cryogenic temperature.

With the enhanced sensitivity, strong intermolecular interactions were detected between chitin and types‐a and type‐d α‐1,3‐glucans, as shown by the unambiguous Ch2‐A^a^3, Ch4‐A^a^3, A^a^3‐Ch4, Ch4‐A^d^3, and A^d^3‐Ch4 cross‐peaks observed in the 100‐ms dipolar‐assisted rotational resonance (DARR) spectrum (Figure 2d). We also observed three cross peaks in a 15‐ms proton‐assisted recoupling (PAR) spectrum, including Ch2‐A^d^1, Ch4‐A^a^3, and A^a^3‐Ch4, further confirming the interactions between chitin and these two specific forms of α‐1,3‐glucans (Figure 2e).^[^ ^49^, ^50^ ^]^ In addition, a cross peak between the carbon‐3 sites of type‐a and type‐d α‐1,3‐glucans (A^a^3‐A^d^3) was detected (Figure 2e). Together, these observations confirm that type‐d and type‐a α‐1,3‐glucans are associated with each other and further packed with chitin in the rigid domain of the cell wall, while type‐b and type‐c α‐1,3‐glucans are not colocalized with chitin microfibrils.

### Two Structural Forms of α‐1,3‐Glucans Host Melanin Deposition

**Figure 3:**
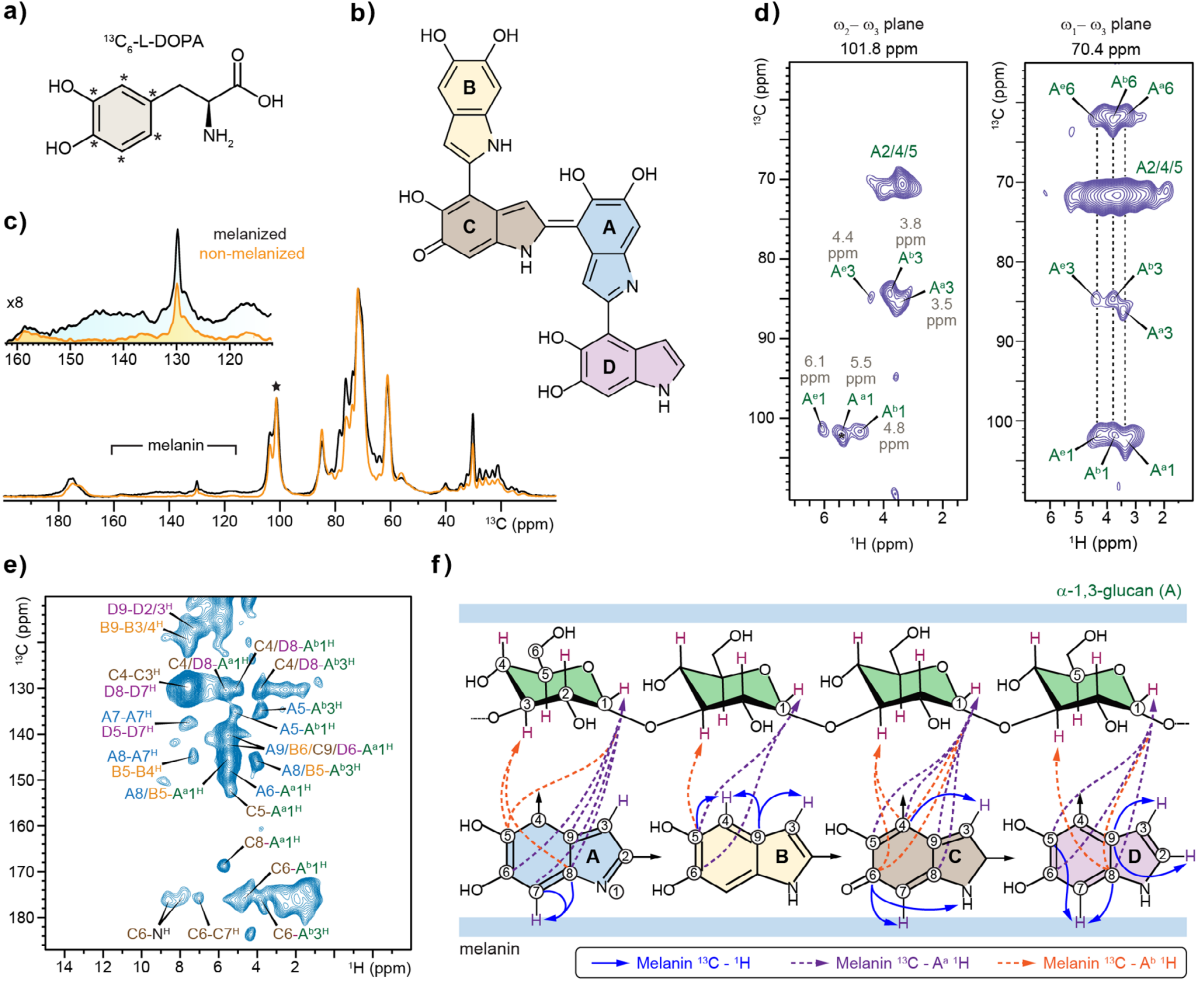
^1^H‐detected solid‐state NMR shows interactions of α‐1,3‐glucan with melanin. a) Structure of melanin precursor ring‐^13^C_6_‐labeled L‐DOPA. Asterisks indicate the ^13^C‐labeling sites. b) Representative structure of DHI melanin formed by covalently linked subunits. c) 1D ^13^C CP spectra of non‐melanized (orange) and melanized (black) *C. neoformans* H99 cells. The spectra are normalized by the α‐1,3‐glucan carbon 1 peak at 101 ppm (asterisk). The zoom‐in region shows the aromatic signals from melanin, with intensities magnified by 8‐fold. d) Polymorphic forms of α‐1,3‐glucans (A^a^, A^b^, and A^e^) resolved using ^1^H‐detected 3D hCCH TOCSY (WALTZ‐16) spectrum. ^1^H chemical shifts (ppm) are in gray. Key strips are extracted for *ω*_2_–*ω*_3_ (^13^C‐^1^H) and *ω*_1_–*ω*_3_ (^13^C‐^1^H) planes. e) 2D hChH with 0.8 ms RFDR mixing showing intermolecular interaction between melanin non‐protonated carbons and α‐1,3‐glucan protons. f) Structural summary of observed interactions between cell wall α‐1,3‐glucan and melanin units. Carbon and nitrogen atom numbering in melanin fragments follows the standard convention for indoles. Blue solid lines: cross peaks observed between non‐protonated carbons and protons in protonated sites within melanin. Dashed lines in purple and orange indicate the intermolecular interactions between melanin carbons and protons in type‐a and type‐b α‐1,3‐glucans, respectively. The experiments were performed on 600 MHz NMR at 60 kHz MAS.

In addition to the capsule, another crucial virulence factor of *Cryptococcus* species is their ability to synthesize melanin, which is deposited in the cell wall, and forms a protective barrier against environmental and host stressors.^[^ ^31^ ^]^ Melanin polymers are proposed to be composed of stacked aromatic and indolic rings, formed via the oxidative polymerization of L‐3,4‐dihydroxyphenylalanine (L‐DOPA) catalyzed by the laccase enzyme (Figure 3a,b).^[^ ^51^, ^52^ ^]^ Since *Cryptococcus* requires an exogenous substrate for melanin biosynthesis, we supplemented the minimal medium with 1 mM ring‐^13^C_6_‐labeled L‐DOPA to cultivate melanin‐rich *C. neoformans* H99 cells.^[^ ^53^, ^54^, ^55^ ^]^ Consequently, the melanin‐labeled cells exhibited enhanced aromatic ^13^C signals in the 110–160 ppm range, particularly in the 140–150 ppm region, corresponding to non‐protonated melanin carbon sites (Figure 3c, see expanded spectral region). These distinct melanin aromatic signals enabled us to establish correlations with carbohydrate proton resonances to map out melanin‐carbohydrate interactions within intact cells.

We further identified three polymorphic forms of α‐1,3‐glucan in melanized *C. neoformans* cells, designated A^a^, A^b^, and A^e^, from the ^13^C‐^1^H strips extracted from the 3D hCCH TOCSY (WALTZ‐16) spectrum (Figure 3d and Table S7). These polymorphs exhibited distinct ^1^H chemical shifts: the anomeric carbon (A1) correlated with three distinct ^1^H resonances at 5.5 ppm (A^a^), 4.8 ppm (A^b^), and 6.1 ppm (A^e^) in the *ω*_2_–*ω*_3_^13^C‐^1^H strip, while the C3 position correlated with protons at 3.5 ppm (A^a^), 3.8 ppm (A^b^), and 4.4 ppm (A^e^) (Figure 3d). The *ω*_1_–*ω*_3_^1^H‐^13^C strip extracted from 70.4 ppm further revealed the through‐bond carbon connectivity of these three polymorphic forms and established correlations with distinct carbon sites, such as the resolvable carbons at positions 3 and 1 (Figure 3d). In this analysis, the A^c^ and A^d^ forms detected in ^13^C‐based experiments were not observed due to their semi‐dynamic nature or low abundance.

Next, we measured a 2D hChH spectrum, which employed an extended, 0.8 ms radio frequency driven recoupling (RFDR) mixing period to facilitate long‐range ^1^H‐^1^H polarization transfer (Figure 3e). Notably, this experiment facilitated the observation of extensive correlations between non‐protonated carbons and protons at protonated sites within the melanin structure (Figure 3e; blue solid lines in Figure 3f). The ^13^C and ^1^H chemical shifts identified in melanin are summarized in Figure S9 and Tables S8 and S9. In melanin subunit D, key cross‐peaks include those between carbon‐9 and protons‐2/3 (D9‐D2/3^H^), as well as D5‐D7^H^ and D8‐D7^H^ (Figure 3e,f). Similar cross‐peaks were observed within other melanin subunits, such as A7/8‐A7^H^, B9‐B3/4^H^, B5‐B4^H^, C4‐C3^H^, and C6‐C7^H^. The C6‐N^H^ cross‐peak further revealed spatial proximity between C‐fragment carbons and indole amide protons from the same or neighboring subunits.

Importantly, the hChH experiment simultaneously revealed extensive intermolecular interactions between the carbons in the indole rings of melanin and the protons in carbohydrates. For example, the carbon 4 of melanin subunit‐C and carbon 8 of subunit‐D, two non‐protonated carbons resonating at 130 ppm, exhibited correlations with the proton 1 of type‐a α‐1,3‐glucan at 5.5 ppm, the proton 1 of type‐b α‐1,3‐glucan at 4.8 ppm, and the proton 3 of type‐b α‐1,3‐glucan at 3.8 ppm, resulting in the C4/D8‐A^a^1^H^, C4/D8‐A^b^1^H^, and C4/D8‐A^b^3^H^ cross peaks (Figure 3e). Type‐a and type‐b α‐1,3‐glucans showed 13 and 10 strong cross‐peaks with melanin, respectively, including A8/B5‐A^a^1^H^, A5‐A^b^3^H^, A5‐A^b^1^H^, A6‐A^b^1^H^, C5‐A^a^1^H^, C8‐A^a^1^H^, C6‐A^b^1^H^, and C6‐A^b^3^H^, in addition to those previously described (Figure 3e,f). These intermolecular interactions provide the first molecular‐level experimental evidence that α‐1,3‐glucans act as a previously unrecognized partner with melanin, with their type‐a and type‐b structural variants serving as the primary interactors.

## Discussion

Recent advances in solid‐state NMR spectroscopy have significantly enhanced our ability to investigate fungal cell walls, offering detailed insights into the structures of wall polymers, as well as their hydration, dynamics, and intermolecular interactions.^[^ ^56^, ^57^, ^58^, ^59^, ^60^ ^]^ Such analyses describe molecular behavior in the context of intact cells, in a way that prior methods, which relied on physical, chemical, and/or enzymatic perturbation, could not.^[^ ^61^, ^62^ ^]^ The application of these techniques to the major fungal pathogen *C. neoformans* in this study has uncovered unexpected features of the cell wall and revealed its interactions with two critical virulence factors: the extracellular capsule and cell‐wall‐associated melanin.

**Figure 4:**
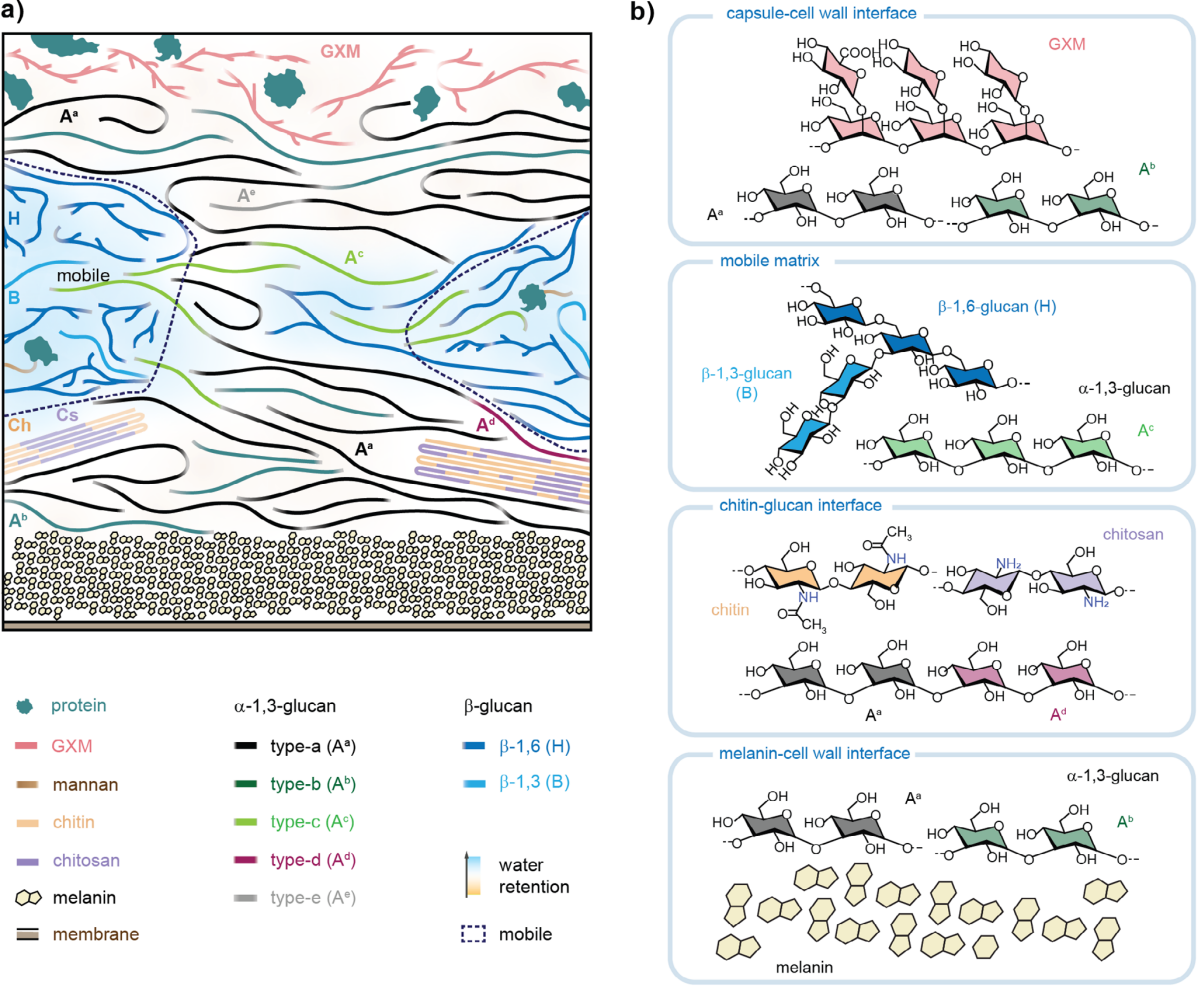
Organization of cryptococcal cell walls and association with capsule and melanin. a) Structural scheme based on NMR data from H99 cells, integrated with previously reported biochemical and imaging analyses.^[^ ^16^, ^31^, ^63^ ^]^ Five distinct forms of α‐1,3‐glucans constitute 70% of the rigid polysaccharides. Capsular polysaccharides, primarily GXM, are associated with type‐a and type‐b α‐1,3‐glucans in the cell wall. Melanin is stabilized by type‐a and type‐b α‐1,3‐glucans. Molecules associated with type‐a and type‐b α‐1,3‐glucans are rigidified and dehydrated. Chitin and chitosan, which together account for only 6% of rigid molecules, exhibit a high deacetylation level of approximately 30% and are stabilized through interactions with type‐d and type‐a α‐1,3‐glucans, both of which colocalize. The mobile phase (highlighted by dashed lines) is predominantly composed of β‐1,6‐glucan (70%), which regulates the water association of cryptococcal cell wall, with a minor fraction of β‐1,3‐glucan, present either as linear chains or as sidechains of β‐1,6‐glucan. Additionally, type‐c α‐1,3‐glucan extends through rigid and mobile phases. Type‐e α‐1,3‐glucan represents a minor structural variant that does not exhibit an association with melanin. Molecular fractions are considered in this model, though the representation is not strictly to scale. The background gradient illustrates the water distribution within the cell wall. b) Structural illustration of molecules residing on key intermolecular interfaces or structural domains in *C. neoformans*.

Previous studies of cryptococcal cell walls, based on structural and imaging analyses, suggested a bilayer structure with an inner layer composed of interlinked chitin, chitosan, and β‐glucans and an outer layer composed of α‐ and β‐glucans.^[^ ^16^, ^17^, ^63^ ^]^ In these models, melanin deposition occurs initially at the inner portion of the cell wall near the plasma membrane, and later builds up until it is more thoroughly distributed; the capsule polysaccharide is associated with α‐1,3‐glucan in the outer layer.^[^ ^23^, ^25^ ^]^ The NMR‐revised model presented in Figure 4 highlights the broad distribution, complex structures, and diverse structural functions of α‐1,3‐glucan in maintaining cell wall integrity and identifies this molecule as a potential target for antifungal therapy against cryptococcal meningitis, as disruption of α‐1,3‐glucan synthesis results in avirulent cells.^[^ ^25^ ^]^ The model also explains the persistent association between melanin and the cell wall, even in samples with relatively low chitin and chitosan content.^[^ ^16^ ^]^

The integration of solid‐state NMR data with existing chemical and imaging analyses has introduced three novel structural concepts that redefine the structural paradigm of the cryptococcal cell wall (Figure 4). First, our NMR data highlights the role of α‐1,3‐glucans, the most abundant polysaccharide in *C. neoformans* cell walls, as key structural components, present both in the inner wall, where it interacts with chitin and initially developing melanin, and in the outer wall, where it promotes capsule attachment. This resolves a long‐standing knowledge gap concerning the association between the cell wall and two key virulence factors: melanin and capsule. Second, we demonstrate that the broad distribution and functional diversity of α‐1,3‐glucans in *C. neoformans* correlate with the presence of five distinct α‐1,3‐glucan morphotypes, resolved by ^13^C and ^1^H solid‐state NMR in combination with DNP‐enhanced sensitivity. Third, we found that β‐1,6‐glucan, an under‐investigated component in the fungal cell wall,^[^ ^64^ ^]^ is distributed across dynamically distinct domains. It is primarily localized in the mobile phase, where it forms the main component of the soft matrix, with smaller fractions extending into the rigid phase to contribute to structural integrity. This diverse dynamics may also help to explain the limited efficacy of echinocandins against *Cryptococcus* species, as β‐1,3‐glucan is present in low abundance and is confined exclusively to the mobile phase, while β‐1,6‐ and α‐glucans otherwise dominate the wall.

Notably, the cell wall α‐1,3‐glucan of *C. neoformans* occurs in five distinct morphotypes, which we were able to resolve using high‐resolution ^13^C solid‐state NMR, combined with advanced ^1^H detection and DNP sensitivity enhancement (Figure 4). Type‐a and type‐b α‐1,3‐glucans are the predominant structured forms, comprising 55%–75% of all rigid polysaccharides (Figure 1e). These forms aggregate into partially dehydrated bundles, facilitating capsule deposition on the cell wall surface and rigidifying the capsule polysaccharide GXM (Figure 2a–c; upper portion of Figure 4). This highlights the essential role of α‐1,3‐glucan in capsule‐cell wall attachment, and provides a structural explanation for why disruption of the α‐1,3‐glucan synthase gene results in *C. neoformans* cells lacking a surface capsule, despite the successful production of capsule components.^[^ ^65^ ^]^ The dominance of α‐1,3‐glucan we observed in the cell walls of both serotypes also explains the dramatic wall perturbation observed in such mutants, and their poor growth.^[^ ^66^ ^]^

Surprisingly, the major forms of α‐1,3‐glucan (types a and b) that interact with capsule polysaccharide are also situated in a location that makes them capable of hosting melanin deposition (Figure 3e; bottom portion of Figure 4). These unexpected findings lead to two novel conclusions. First, they identify a previously unrecognized wall component that could organize melanin deposition. Despite the well‐established role of chitin and chitosan in *C. neoformans* melanin deposition, the potential involvement of α‐1,3‐glucan was not known even though the cell wall is implicated in this process.^[^ ^29^, ^51^, ^54^ ^]^ Second, they indicate that these two forms of α‐1,3‐glucans exhibit a broad spatial distribution; they are localized both at the cell surface, where they mediate capsule association, and near the plasma membrane, where they colocalize with melanin (Figure 4).

The remaining three forms of α‐1,3‐glucan, though less abundant, are also functionally significant. Type‐c is present in both the rigid and mobile phases (Figure 1e,h), and thus plays a critical role in integrating α‐1,3‐glucan aggregates into the mobile matrix. Type‐d α‐1,3‐glucan is a minor but highly ordered form that colocalizes with type‐a α‐glucan, both of which are physically associated with chitin microfibrils (Figure 2d,e). Type‐e, another minor form, underlies the ^13^C signals of type‐a and type‐b α‐glucans and is distinguishable by ^1^H chemical shifts, indicating local structural variations compared to the predominant forms (Figure 3d).

Recently, five distinct allomorphic forms of α‐1,3‐glucans have been identified in *A. fumigatus* using high‐resolution ^13^C NMR data.^[^ ^67^ ^]^ Of these, two allomorphic forms were observed only after treatment with the antifungal drug caspofungin (Figure S10a). Comparative analysis of chemical shifts revealed that only the type‐a α‐1,3‐glucan in *C. neoformans* exhibited high structural similarity to some of the *A. fumigatus* forms, with low RMSD values of 0.5–0.6 ppm in chemical shift deviations (Figure S10b).^[^ ^68^ ^]^ This structure also represents the most abundant form of bulk α‐1,3‐glucans identified in both fungal species. In contrast, the remaining four forms in *C. neoformans* (types b–e) displayed distinct chemical shift profiles, with higher RMSD values of 1.0–3.0 ppm, indicating that they represent distinct structures for this molecule.

Our results provide an unprecedented understanding of the structural complexity of α‐1,3‐glucan in the construction of cryptococcal cell walls and highlight its association with two key virulence factors anchored at opposite aspects of the cell wall. The multiple roles of this versatile polymer may also explain its unusually high abundance in *Cryptococcus* species. Meanwhile, these findings raise new questions. It remains unclear whether the observed structural forms result from complexity in α‐1,3‐glucan biosynthesis and whether they are universally present across different *Cryptococcus* species, other serotypes, and other pathogenic fungal species. It also remains to be determined whether α‐1,3‐glucan synthases or other enzymes involved in biosynthesis and modification could be developed as therapeutic targets. Addressing these questions would advance our understanding of cryptococcal cell wall biosynthesis and structure, providing molecular insights that are essential for the development of effective antifungal therapies against cryptococcosis.

## Supporting Information

The authors have cited additional references within the Supporting Information.^[^ ^69^, ^70^, ^71^, ^72^, ^73^, ^74^, ^75^, ^76^, ^77^, ^78^, ^79^, ^80^, ^81^, ^82^, ^83^, ^84^, ^85^, ^86^, ^87^, ^88^, ^89^, ^90^, ^91^ ^]^

## Conflict of Interests

The authors declare no conflict of interest.

## Supporting information



Supporting Information

## Data Availability

The data that support the findings of this study are available from the corresponding author upon reasonable request.
