# Supplementary material for: Polymorphic α‐Glucans as Structural Scaffolds in Cryptococcus Cell Walls for Chitin, Capsule, and Melanin: Insights From 13C and 1H Solid‐State NMR
Source: Angew Chem Int Ed Engl. 2025 Jul 15;64(36):e202510409. doi: 10.1002/anie.202510409 (PMC12366749; doi:10.1002/anie.202510409)
Supplement: Supplementary file 1 — Supporting Information [file ANIE-64-e202510409-s001.pdf]

Supporting Information

Polymorphic α-Glucans as Structural Scaffolds in *Cryptococcus* Cell Walls for Chitin, Capsule, and Melanin: Insights from ^{13}C and ^{1}H Solid-State NMR

Ankur Ankur[1#], Jayasubba Reddy Yarava[1#], Isha Gautam[1], Faith J. Scott[2], Frederic Mentink-Vigier[2,3], Christine Chrissian[4,5], Li Xie[1], Dibakar Roy[1], Ruth E. Stark[4,5], Tamara L. Doering[6], Ping Wang[7], Tuo Wang[1,*]

[1] Department of Chemistry, Michigan State University, East Lansing, MI, USA

[2] National High Magnetic Field Laboratory, Florida State University, Tallahassee, FL, USA

[3] Department of Chemistry and Biochemistry, Florida State University, Tallahassee, FL, USA

[4] Department of Chemistry and Biochemistry, City College of New York, New York, NY, USA

[5] CUNY Institute for Macromolecular Assemblies, The City University of New York, New York, NY, USA

[6] Department of Molecular Microbiology, Washington University in St. Louis School of Medicine, St. Louis, MO, USA

[7] Departments of Microbiology, Immunology & Parasitology, Louisiana State University Health Sciences Center, New Orleans, LA, USA

[#] These authors contributed equally

[*] Correspondence: wangtuo1@msu.edu

EXPERIMENTAL SECTION

Preparation of ^{13}C,^{15}N-labeled fungal material

C. neoformans strains H99, JEC20, *pka1*, and Cap70 were cultured in ^{13}C, ^{15}N-enriched growth media. The growth medium consisted of 1.7% Yeast Nitrogen Base (YNB), 2.1% ^{13}C-glucose (Cambridge Isotope Laboratories, CLM-1396-PK), and 1.5% ^{15}N-ammonium sulfate (Cambridge Isotope Laboratories, NLM-713-PK), with the pH adjusted to 5.5 using 1M HEPES buffer. Cells were cultured in 100 mL liquid medium in 250-mL Erlenmeyer flasks and incubated at 37°C with shaking at 150 rpm (1364 × g). Fungal biomass was harvested by centrifugation at 4,000 rpm (13,700 × g) for 5 min at 4°C and the pellet was washed four times using nano-purified water followed by the centrifugation procedures. For solid-state NMR characterization, 35-45 mg of natively hydrated whole-cell material was packed into a 3.2-mm magic-angle spinning (MAS) rotor (Cortecnet, HZ16916).

To generate melanin-rich *C. neoformans* cells, H99 cells was incubated for 10-14 days at 30°C with shaking at 150 rpm in a minimal medium (29.4 mM KH_2PO_4, 10 mM $MgSO_4$, 13 mM glycine, 3 μM thiamine, 15 mM ^{13}C-glucose, pH 5.5) supplemented with 1 mM $^{13}C_6$-L-DOPA (Cambridge Isotope Laboratories, CLM-1007-PK)[53, 54]. Melanized fungal cells were collected and washed following the protocol described above and packed into 3.2 mm and 1.3 mm MAS rotors (Cortecnet, HZ14752) for solid-state NMR characterization.

Scanning electron microscopy

A small amount of the same *C. neformans* material used for NMR analysis were suspended in distilled water, mixed with 4% glutaraldehyde in 0.1 M sodium phosphate buffer (pH 7.4), and fixed for 30 min at 4°C. To prepare coverslip-mounted samples, 1% Poly-L-Lysine (Sigma Aldrich, P1399) was applied as a single drop onto a plastic Petri dish, over which a 12 mm round glass coverslip was placed and allowed to adhere for 10 min. The coverslip was then removed, gently rinsed with water, and drained while preventing complete drying. A drop of the fixed cell suspension was placed on the previously poly-lysine-exposed surface of the coverslip and allowed to settle for 10 minutes. The sample was subsequently rinsed with water and dehydrated through a graded ethanol series (25%, 50%, 75%, 95%) for 10 min at each step, followed by three 10-min changes in 100% ethanol and critical point drying using a Leica Microsystems EM CPD300 critical point dryer (Leica Microsystems, Vienna, Austria) with carbon dioxide as the transitional fluid. Dried samples were mounted on aluminum stubs, sputter-coated with osmium, and examined using a JEOL 7500F field emission scanning

electron microscope operating at 15 kV. Total cell diameter was quantified using ImageJ software (version V1.8.0_172) and reported in **Table S1**.

^{13}C solid-state NMR experiments

High-resolution 1D and 2D solid-state NMR experiments were conducted at 800 MHz (18.8 Tesla) at Michigan State University. All ^{13}C-detection experiments were performed using a 3.2 mm HCN probe at 15 kHz MAS, with ambient temperatures between 283 K and 298 K. ^{13}C chemical shifts were externally referenced by calibrating the adamantane CH_2 peak to 38.48 ppm, and the resulting spectral reference (sr) value was applied to fungal spectra. Unless otherwise specified, typical radiofrequency field strengths were 83-100 kHz for ^{1}H decoupling, 62.5 kHz for ^{1}H hard pulses, and 50-62.5 kHz for ^{13}C. Experimental parameters for all NMR spectra are documented in **Table S10**.

1D ^{13}C spectra were acquired using various polarization methods to probe molecular dynamics. Rigid components were detected via dipolar-based ^{13}C cross-polarization (CP) with a 1-ms contact time, adhering to Hartmann-Hahn match conditions of 62.5 kHz for ^{13}C and ^{1}H. For quantitative analysis, 1D ^{13}C direct polarization (DP) experiments were conducted with a long recycle delay of 35 s, ensuring full longitudinal relaxation before the next scan. Additionally, a shorter recycle delay of 2 s in the same ^{13}C DP experiment enabled selective detection of mobile molecules with fast ^{13}C-T_1 relaxation. These 1D experiments were performed on three independently prepared *C. neoformans* sample replicates, demonstrating high reproducibility (**Figure S11**).

To facilitate resonance assignment, 2D ^{13}C-^{13}C correlation experiment was conducted using a 53-ms CORD (combined $R2_n^v$-driven) mixing period, which revealed intramolecular cross-peaks between carbon sites within each molecule (**Figure S1**)[43]. Additionally, 2D ^{13}C refocused J-INADEQUATE experiments were conducted using either CP or DP (1.5 s recycle delay) to probe molecular domains with differing mobility (**Figure S4**)[69]. This experiment correlated double-quantum (DQ) chemical shifts with two corresponding single-quantum (SQ) shifts, generating asymmetric spectra that enabled efficient through-bond carbon-connectivity tracking. To optimize carbohydrate detection, the τ period (out of four) was set to 2.3 ms. All 1D experiments, as well as 2D CORD experiments, were conducted for all *C. neoformans* strains. Assigned chemical shifts for rigid and mobile polysaccharides are documented in **Table**

S2. Data acquisition was conducted using Topspin 3.5, and spectral analysis was performed in Topspin 4.0.8. Figures were prepared using Adobe Illustrator CS6 (V16.0.0).

Molecular composition analysis was conducted by selecting only well-resolved signals in 2D ^{13}C CORD spectra for rigid components and ^{13}C DP refocused J-INADEQUATE spectra for mobile molecules (**Tables S3** and **S4**). Peak volumes were quantified using the integration function in Bruker Topspin, with quantification based on the mean of resolved signals. Relative polysaccharide abundance was determined by normalizing the sum of integrals with their respective counts[70]. The standard error for each polysaccharide was calculated by dividing the standard deviation of integrated peak volumes by the total cross-peak count. The overall standard error was computed as the square root of the sum of squared errors for each polysaccharide. The percentage error was determined by normalizing the standard error with the average integrated peak volume and adjusting for each polysaccharide's relative abundance[70].

Solid-state NMR of polymer hydration and dynamics

All polymer hydration and dynamics experiments were conducted on a Bruker Avance Neo 400 MHz (9.4 T) NMR spectrometer at Michigan State University using a 3.2 mm HCN MAS Bruker probe at 280 K. Water accessibility of polysaccharides was assessed using 1D and 2D water-edited ^{13}C-^{13}C correlation spectra[45, 47]. A ^{1}H-T_2 filter (0.6-1.2 ms, strain-dependent) was applied to suppress carbohydrate signals to less than 10% but preserve 82-88% of water magnetization. (**Figure S6**), followed by ^{1}H-^{1}H mixing to transfer water ^{1}H magnetization to hydrated carbohydrates. ^{13}C detection was achieved via CP with a 1-ms contact time. For 2D water-edited experiments, a 4 ms ^{1}H mixing period and 50 ms DARR mixing were used. Intensity ratios (S/S_0) between the water-edited (S) and control (S_0) spectra were obtained to quantify water retention around each carbon site (**Table S5**).

^{13}C-T_1 relaxation was measured using the Torchia-CP scheme[71] with z-filter durations from 0.1 μs to 8 s. For ^{13}C-detected ^{1}H-$T_{1\rho}$ relaxation, a Lee-Goldburg (LG) spinlock sequence combined with LG-CP suppressed ^{1}H spin diffusion, enabling site-specific measurements via bonded ^{13}C detection[72, 73]. For both measurements, peak intensity decay was fitted to a single exponential equation to determine the corresponding relaxation time constants (**Figures S7, 8** and **Table S6**). The analysis was conducted using OriginPro 9.

Proton-detection solid-state NMR experiments

2D hCH, 2D hChH, and 3D hCCH TOCSY correlation experiments were conducted using a 600 MHz Bruker Avance Neo spectrometer at Michigan State University, equipped with a 1.3 mm triple-resonance MAS probe. Samples were spun at 60 kHz MAS. The 2D hChH experiment employed a 0.8 ms RFDR-XY16 (radiofrequency-driven recoupling) mixing[74, 75]. A total of 192 time-domain (TD) points were acquired for the indirect dimension, with 512 transients co-added per TD and a recycle delay of 2.0 s. The total experimental duration was 58 hr. A 2D hCH spectrum was acquired on the same 600 MHz Bruker spectrometer under identical conditions, but without RFDR mixing, to enable direct comparison. The hCH experiment were measured using a short second-CP of 100 μs. The 2D data were collected using the States-TPPI method[76]. Through-bond ^{13}C-^{13}C correlations were established using the 3D hCCH TOCSY (total correlation spectroscopy) experiment[77], employing a 15 ms WALTZ-16 (wideband alternating-phase low-power technique for zero-residual splitting) mixing period at an rf field strength of 21.4 kHz[78]. A total of 128 × 128 TD points were acquired in the t_1 and t_2 evolution periods, with 8 transients co-added per TD point. The total experimental time was 78 h.

The Heteronuclear dipolar decoupling sequence slpTPPM (swept low-power two-pulse phase modulation)[79] was applied during the t_1 evolution period for the 2D hCH and 2D hChH sequences, and during both t_1 and t_2 periods in the 3D hCCH TOCSY experiment, with a rf field strength of 21.0 kHz. For the direct ^1H detection period, WALTZ-16 decoupling was applied on the ^{13}C channel with a rf field strength of 12.5 kHz for 2D hCH and 2D hChH, and at 21.4 kHz for 3D hCCH TOCSY. Water suppression was achieved using the MISSISSIPPI (multiple intense solvent suppression intended for sensitive spectroscopic investigation of protonated proteins) sequence[80] on the ^1H channel, with a rf of 15.2 kHz applied for 100 ms. The actual sample temperature was measured to be 304 K, based on the ^1H chemical shift of water relative to the DSS signal at 0 ppm. ^1H and ^{13}C chemical shifts of *C. neoformans* polysaccharides, as well as ^{13}C chemical shifts of melanin fragments detected in ^1H-detected experiments, were documented in **Tables S7** and **S8**. Experimental details are summarized in **Table S11**.

MAS-DNP analysis of inter-polysaccharide interactions

A stock solution containing 10 mM AsymPol-POK bi-radicals in a d_6-DMSO/H_2O (10/90 vol%) mixture was prepared[48]. ^{13}C, ^{15}N-labeled *C. neoformans* H99 cells were mixed with 50

μL of the stock solution and gently ground using a set of mortar and pestle to ensure radical penetration and distribution into the porous cell walls. Approximately 30 mg of the processed sample was packed into a 3.2-mm sapphire rotor for DNP experiments. All experiments were conducted on a 600 MHz/395 GHz MAS-DNP spectrometer at the National High Magnetic Field Laboratory (Tallahassee, FL, USA), equipped with an 89 mm bore and a gyrotron microwave source. Data acquisition utilized a 3.2-mm HCN probe operating at 8 kHz MAS and 100 K. The gyrotron cathode current was maintained between 130-150 mA, with a voltage setting of 16.2 kV. The power of the microwave irradiation was 6.5 W. The NMR sensitivity enhancement ($\varepsilon_{on/off}$) was 11, and the DNP signal buildup time was ~2.9 s for *C. neoformans* carbohydrate signals. The PAR spectrum was acquired with 15 ms recoupling duration, during which the ^{1}H and ^{13}C irradiation frequencies were set at 53 kHz and 50 kHz, respectively[49, 50]. The 2D ^{13}C-^{13}C DARR spectrum was recorded with a 100 ms mixing time.

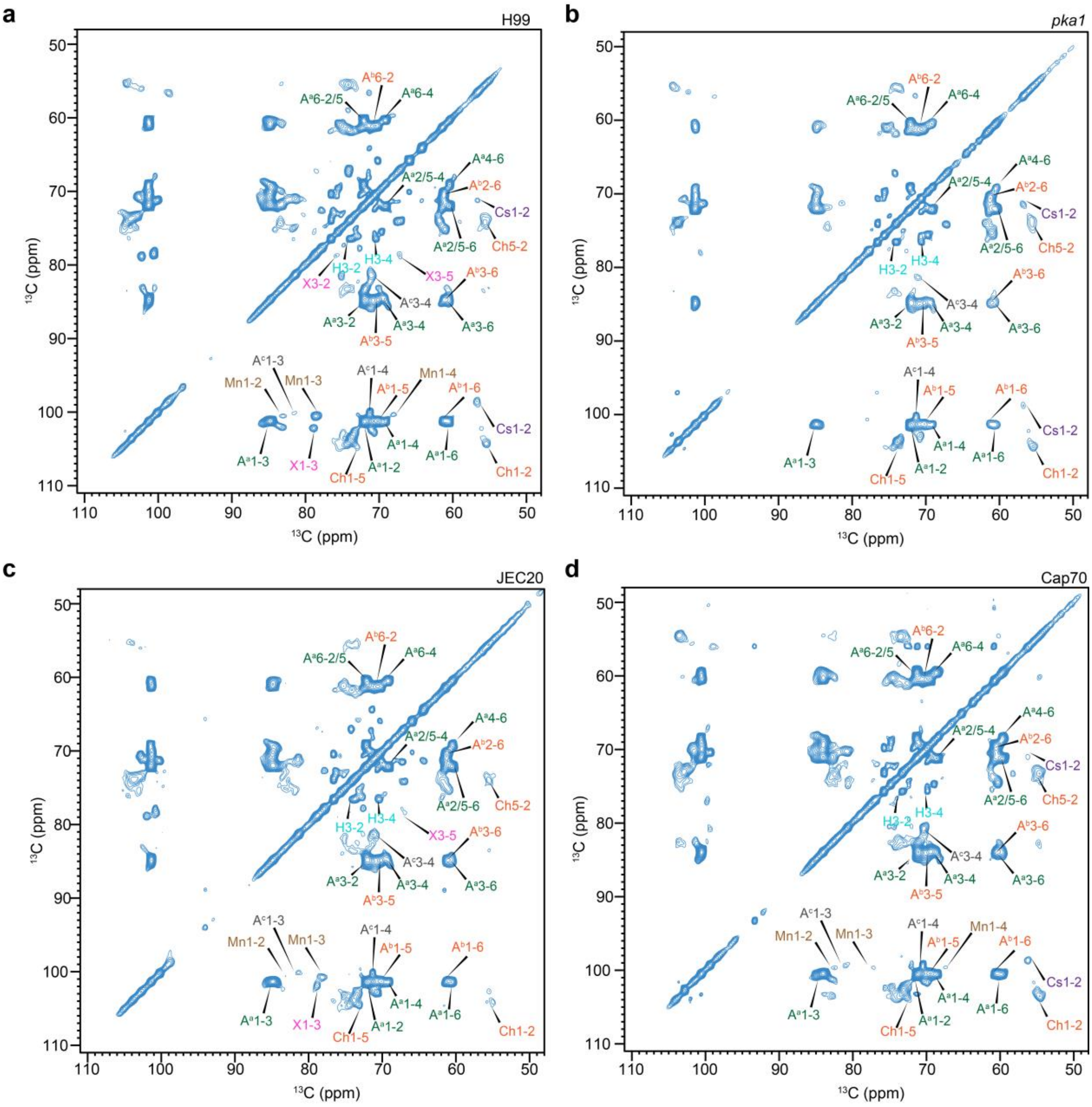

Figure S1. Resonance assignment of rigid glucans in *C. neoformans* cell wall. CP-based 2D ^{13}C-^{13}C correlation spectrum measured with 53 ms CORD mixing for **(a)** H99, **(b)** *pka1*, **(c)** JEC20, and **(d)** Cap70.

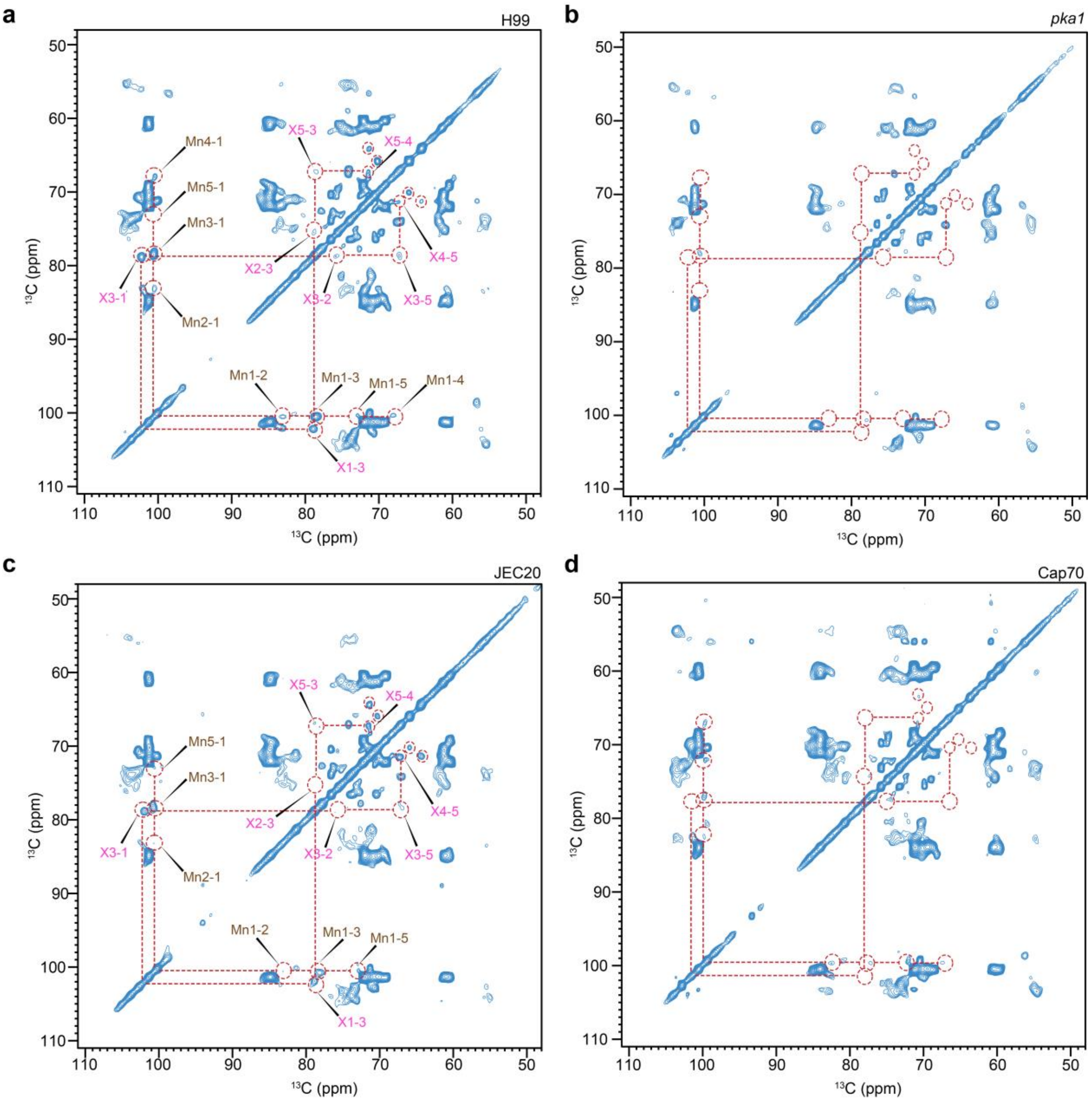

Figure S2. Resonance assignment of capsular molecules in *C. neoformans*. (a) CP-based 2D ^{13}C-^{13}C correlation spectrum measured with 53 ms CORD mixing for H99, highlighting mannan and xylose signals arising from capsules. **(b)** CP-based 2D ^{13}C-^{13}C correlation spectrum measured with 53 ms CORD mixing for *pka1* highlighting the absence of mannan and xylose signals. **(c)** CP-based 2D ^{13}C-^{13}C correlation spectrum measured with 53 ms CORD mixing for JEC20 highlighting mannan and xylose signals arising from capsules. **(d)** CP-based 2D ^{13}C-^{13}C correlation spectrum measured with 53 ms CORD mixing for Cap70 highlighting the presence of mannan and the absence of xylose signals.

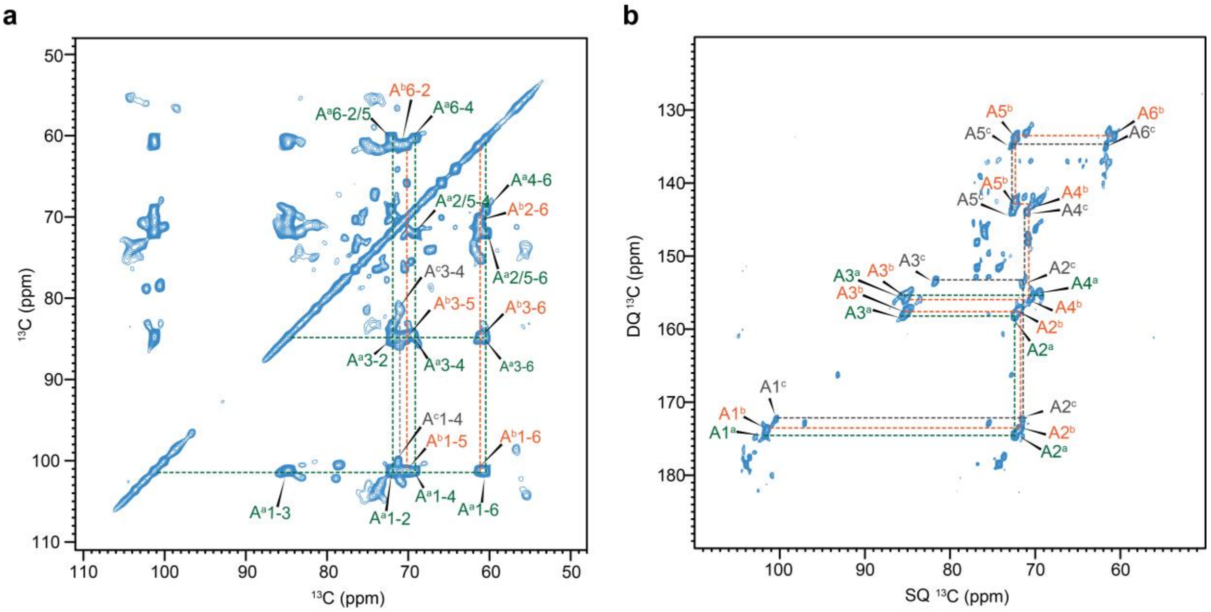

Figure S3. Resonance assignment of α-1,3-glucan in *C. neoformans* cell wall. (a) CP-based 2D ^{13}C-^{13}C correlation spectrum measured with 53 ms CORD mixing. **(b)** Through-bond carbon connectivity was resolved using 2D ^{13}C CP refocused J-INADEQUATE spectrum. Multiple sets of conformers were resolved for α-1,3-glucan. Each peak is annotated with the abbreviation of the carbohydrate name, the subtype (in superscript), and the carbon number. For instance, A^c1 represents the carbon 1 of type-c α-1,3-glucan.

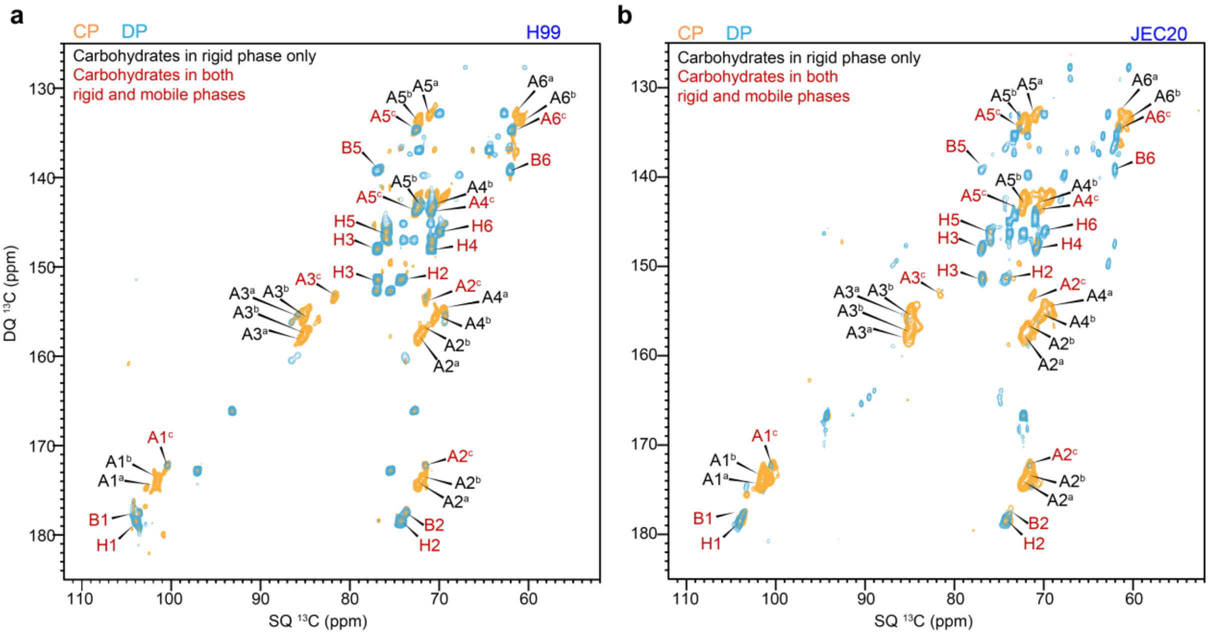

Figure S4. Distribution of α-1,3-glucan, and β-glucan in rigid and mobile domains. Overlay of 2D refocused J-INADEQUATE spectra measured with CP (orange) and DP (cyan) for **(a)** *C. neoformans* H99 and **(b)** *C. neoformans* JEC20 samples. The carbohydrates observed only in the CP-based spectra are rigid and are marked in black. The carbohydrates observed in both CP (INADEQUATE here and CORD in Figure S1) and DP-based spectra are marked in red: these carbohydrates have two-modal distribution in rigid and mobile phases. β-1,6-glucan, β-1,3-glucan, and types-c α-1,3-glucan were observed in both domains for both samples.

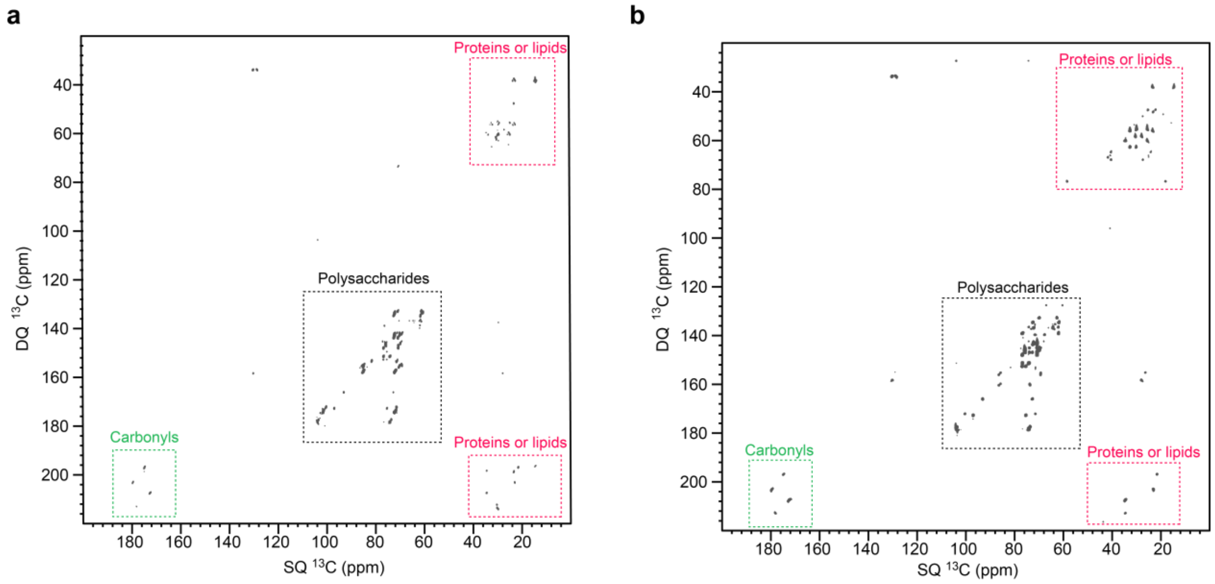

Figure S5. Biopolymer signals resolved in 2D ^{13}C refocused J-INADEQUATE. (a) Rigid components of the cell wall in *C. neoformans* sample probed using 2D CP ^{13}C refocused J-INADEQUAT E spectrum. The individual regions were marked with quadrangles of different colors: green for carbonyls, black for polysaccharides, and red for aliphatics from proteins and lipids. **(b)** Mobile components of *C. neoformans* sample detected using 2D DP ^{13}C refocused J-INADEQUATE spectrum. The individual regions are marked using the same color code mentioned above.

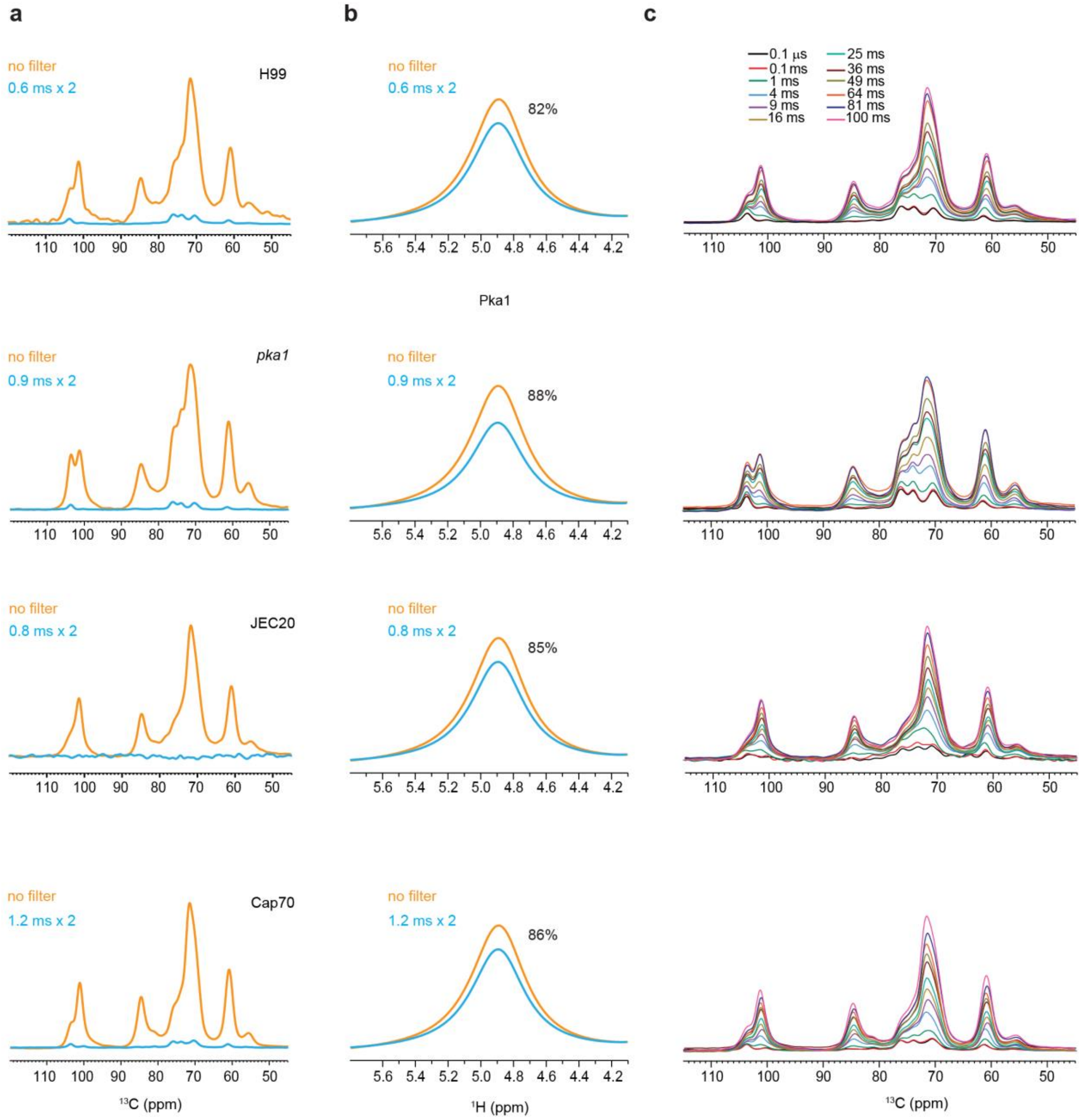

Figure S6. Water-edited experiment setup for inspecting carbohydrate hydration. (a) ^{1}H-T_2 filtered (blue) and control (orange) ^{13}C spectra are shown for four strains. From the top to bottom: H99, *pka1*, JEC20, and Cap70. No spin diffusion was applied. Approximately 85% of carbohydrate ^{13}C signals were removed by the T_2 filter. **(b)** ^{1}H-T_2 filtered (blue) and control (orange) ^{1}H NMR spectra, with 82% and 88% of water signal retained for each strain, after the ^{1}H T_2 filter. From the top to bottom: H99, *pka1*, JEC20, and Cap70. **(c)** 1D water-edited ^{13}C spectra with different ^{1}H mixing times. From the top to bottom: H99, *pka1*, JEC20, and Cap70. All spectra were measured on a 400 MHz spectrometer at 15 kHz MAS at 280 K.

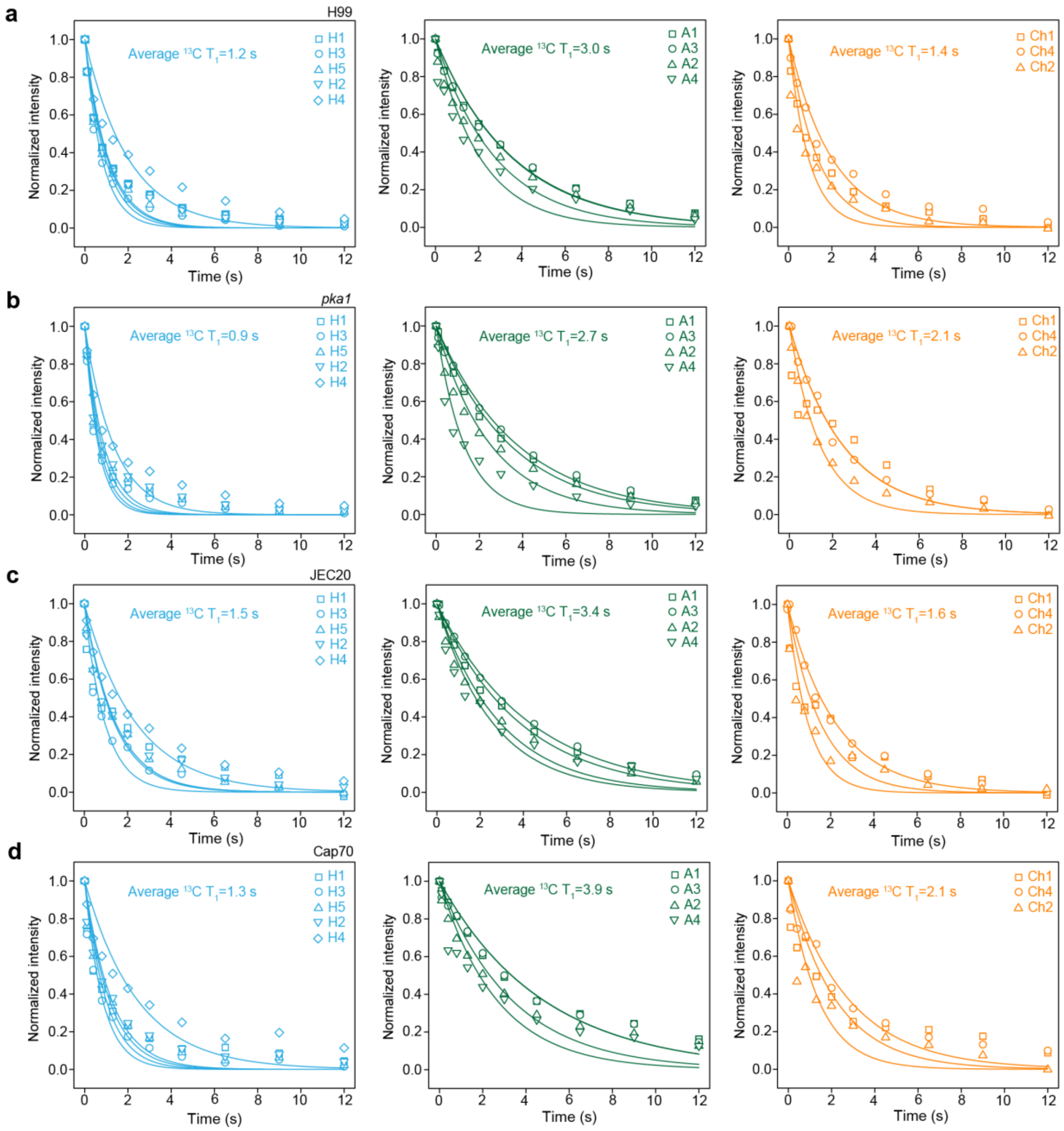

Figure S7. ^{13}C-T_1 relaxation of polysaccharides in *C. neoformans*. ^{13}C-T_1 measured with Torchia CP for **(a)** H99 **(b)** *pka1* **(c)** JEC20 and **(d)** Cap70 samples. The data are separately presented for β-1,6-glucan (light blue), α-1,3-glucan (green), and chitin (orange). The acquired data were fitted to a single exponential decay equation. Different symbols and color codes are used to represent different carbons in these polysaccharides.

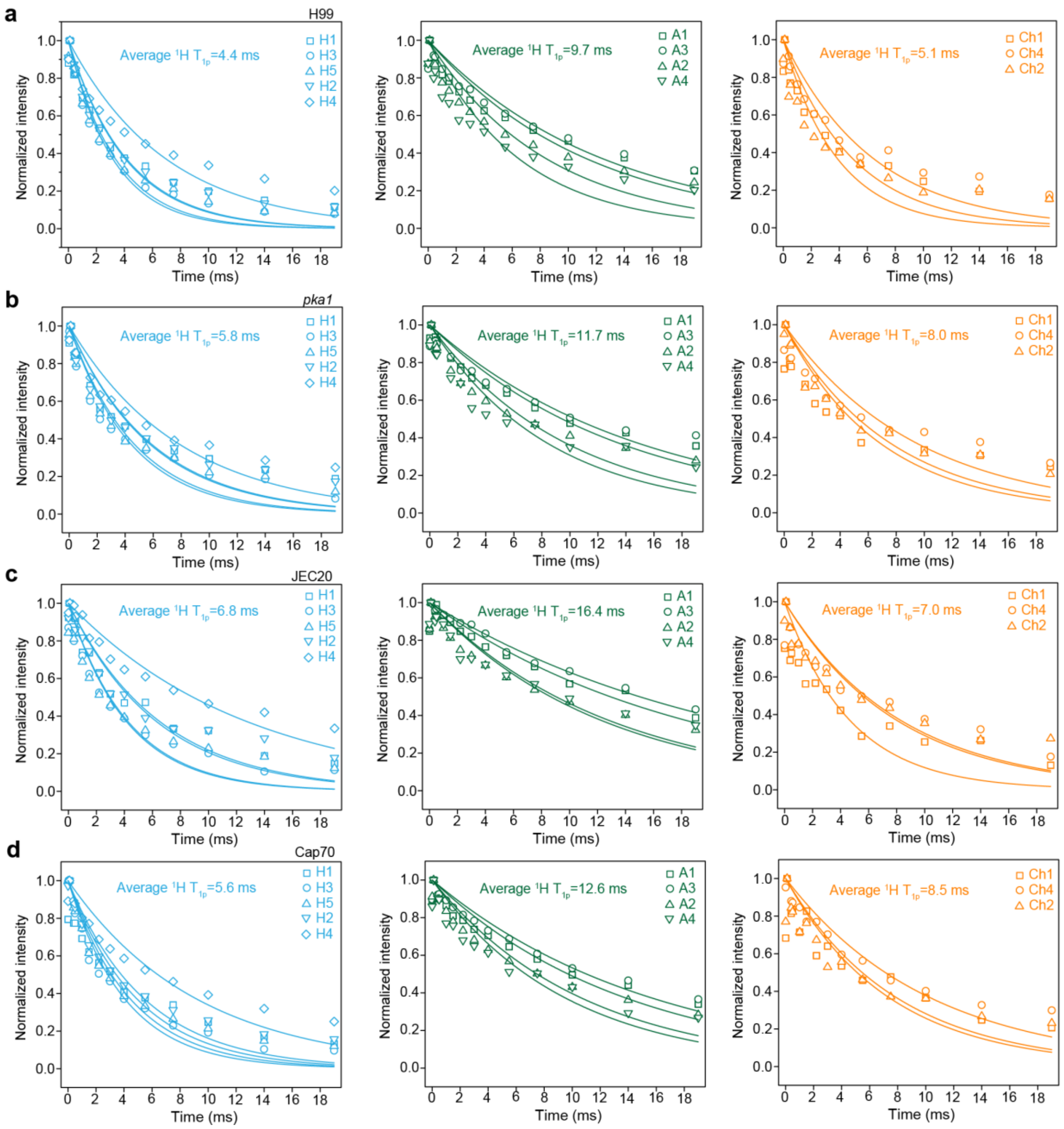

Figure S8. ^{1}H-T$_{1\rho}$ relaxation of polysaccharides in *C. neoformans*. ^{1}H-T$_{1\rho}$ measured with Torchia CP for **(a)** H99 **(b)** *pka1* **(c)** JEC20 and **(d)** Cap70 samples. The data are separately presented for β-1,6-glucan (light blue), α-1,3-glucan (green), and chitin (orange). The acquired data were fitted to a single exponential decay equation. Different symbols and color codes are used to represent different carbons in these polysaccharides.

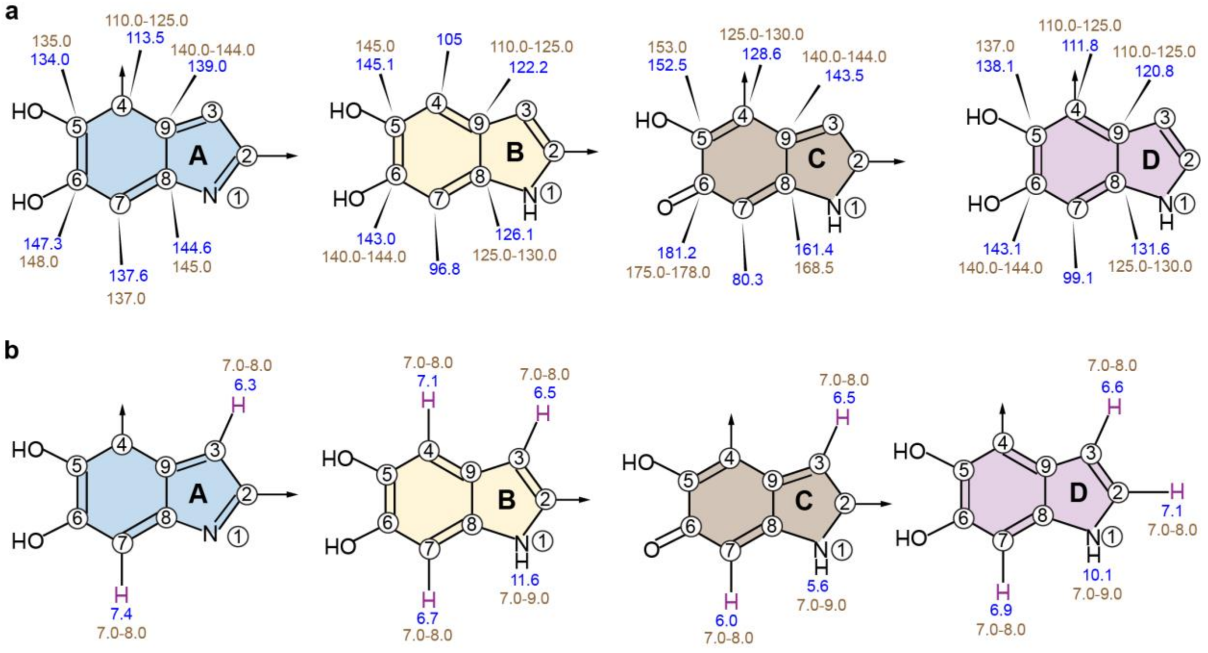

Figure S9. Chemical shifts of melanin fragments in *C. neoformans*. (a) ^{13}C and **(b)** ^{1}H chemical shifts of melanin fragments. Chemical shifts labeled in brown are melanin signals experimentally observed in fast-MAS 2D hChH spectrum. Chemical shifts labeled in blue are predicted by ChemDraw 23.1.1 software.

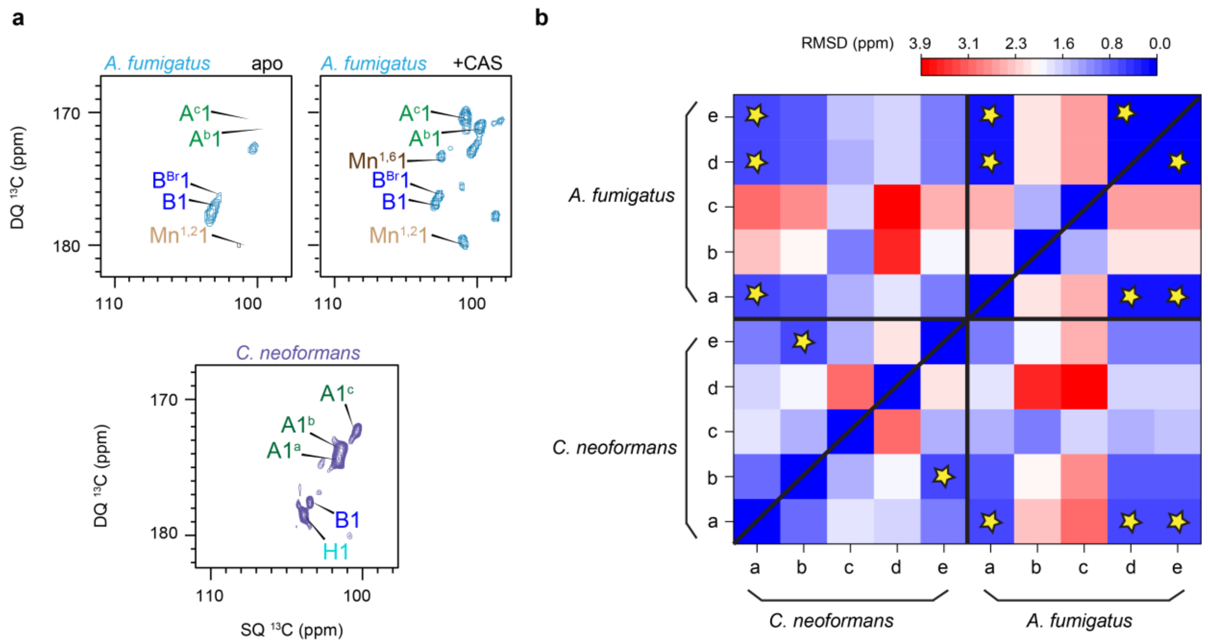

Figure S10. α-1,3-glucans in *Aspergillus fumigatus* and *C. neoformans* cell walls. (**a**) Polymorphic forms of α-1,3-glucans in *A. fumigatus* resolved in 2D refocused J-INADEQUATE spectra measured with DP for apo and caspofungin (CAS)-treated *A. fumigatus*. Upon caspofungin treatment, new polymorphic forms of α-1,3-glucans in *A. fumigatus* (A^b and A^c) were observed. The 2D refocused J-INADEQUATE spectra measured with CP for *C. neoformans* was shown at the bottom. (**b**) Heatmap representation of RMSD for comparison of ^{13}C chemical shifts of different α-1,3-glucan forms identified in *A. fumigatus,* and *C. neoformans*. Asterisks in yellow highlight the pairs of structurally similar forms where the RMSD is below 0.8 ppm, typical for ^{13}C linewidth of fungal polysaccharides.

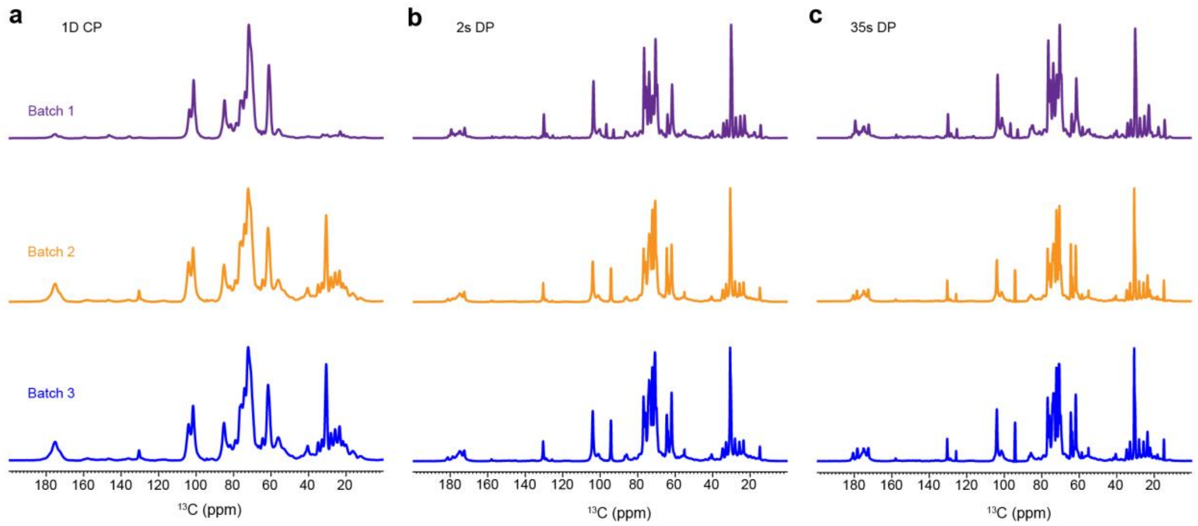

Figure S11. Replication of *C. neoformans* samples. (**a**) 1D ^{13}C CP spectra of *C. neoformans*. (**b**) Mobile components detected by 1D ^{13}C DP with a short recycle delay of 2 s. (**c**) Quantitative ^{13}C DP spectra for the detection of all molecules through a recycle delay of 30 s. The spectra exhibit high reproducibility across three separate batches prepared in 2023 (batch 1), 2024 (batch 2), and 2025 (batch 3). The spectra are highly reproducible except for signals of unused glucose at 93 ppm.

Table S1. The average cell thickness of *Cryptococcus* cells. Results are the average and standard deviation of measurements from different cells (n=24 for H99 and *pka1*, n=19 for JEC20, and n=32 for Cap70). for each sample. A source data file is provided to document each single reading. n=number of cells

Sample	Strain	Average cell thickness (μm)
C. neoformans	H99	5.7±0.5
	pka1	4.6±0.7
	JEC20	5.9±0.6
	Cap70	4.2±0.3

Table S2. ^{13}C chemical shifts of *C. neoformans* polysaccharides in cell walls from ^{13}C-based experiments. The chemical shifts are references on the TMS scale. All chemical shifts are from room-temperature experiments, except for those underlined, which are from DNP-enhanced experiments.

Carbohydrates	form	C1	C2	C3	C4	C5	C6	Reference
Rigid molecules								
α-1,3-glucan (A)	a	101.6<br>101.2	72.4<br>70.6	85.5<br>85.5	69.4<br>/	71.2<br>70.6	61.4<br>61.2	Chakraborty *et al.* 2021[81]
	b	101.4<br>101.0	71.8<br>71.6	85.0<br>84.5	70.5<br>/	72.2<br>/	60.7<br>60.0	
	c	100.4	71.5	81.9	71.1	72.6	61.6	
	d	100.5	70.2	87.6	67.8	70.2	59.9	
β-1,6-glucan (H)		103.8	74.2	76.8	70.8	75.8	69.8	Lowman *et al.* 2011[82]
β-1,3-glucan (B)	a	103.6	73.7	86.5	69.3	76.8	62.0	Chakraborty *et al.* 2021[81]<br>Shim *et al.* 2007[83]<br>Fairweather *et al.* 2009[84]<br>Saito *et al.* 1979[85]
	b	103.6	73.7	85.8	69.3	76.8	62.0	
Chitin		104.3<br>103.4	55.3<br>54.5	73.7<br>73.9	83.0<br>82.5	75.6<br>73.9	62.0<br>62.0	Kang *et al.* 2018[57]<br>Fernando *et al.* 2021[68]
Chitosan		98.4<br>99.1	56.6<br>55.5	/<br>/	/<br>80.2	/<br>/	/<br>60.7	
α-1,2,3-Mannan ($Mn^{1,2,3}$)		100.5	83.0	78.5	68.0	72.8	n.d.	Bacon *et al.* 1995[86]<br>Moyrand *et al.* 2002[87]
Xylose		102.2	75.8	78.8	71.3	67.1	n.a.	
Mobile molecules								
β-1,6-glucan (H)		103.8	74.2	76.8	70.8	75.8	69.8	Lowman *et al.* 2011[82]
β-1,3-glucan (B)	a	103.6	73.7	86.5	69.3	76.8	62.0	Chakraborty *et al.* [81]<br>Shim *et al.* 2007<br>Fairweather *et al.* 2009[84]<br>Saito *et al.* 1979[85]
	b	103.6	73.7	85.8	69.3	76.8	62.0	
α-1,3-glucan (A)	a	/	/	/	/	/	/	
	b	/	/	/	/	/	/	
	c	100.4	71.5	82.0	71.1	72.6	61.6	
α-1,2-Mannan ($Mn^{1,2}$)		101.4	79.2	71.7	67.7	74.3	62.1	Chakraborty *et al.* 2021[81]

Table S3. The molar composition of rigid polysaccharides. The numbers are estimated using integrals (volume) of cross peaks in 2D ^{13}C-^{13}C 53 ms CORD spectra. The average integrals of cross-peaks of each polysaccharide are shown. Error bars are standard errors. Chitosan signals in JEC20 cells have low intensity.

Strain	Polysaccharide							
	α-1,3-glucan			β-1,6-glucan	Chitin	Chitosan	Mannan	Xylose
	a	b	c					
C. neoformans H99	41±13	20±5	9±5	8±2	4±0.7	2±0.6	11±5	5±2
C. neoformans pka1	34±9	21±3	11±5	21±5	8±1.0	5±0.6	/	/
C. neoformans JEC20	38±11	28±8	6±2	7±3	5±2	/	6±3	10±4
C. neoformans Cap70	39±15	36±6	9±4	7±2	5±0.7	2±0.6	2±0.4	/

The area of the following well-resolved cross peaks 53 ms CORD spectra are used:
α-1,3 (a): the average of C1-C2/3/4 and C3-C2/4.
α-1,3 (b): the average of C1-C2/4, C3-2/4.
α-1,3 (c): the average of C1-C2/4, C3-2/4.
β-1,6: the average of C3-C2/4, C5-C4/6.
Chitin: the average of C1-2/5, C5-C2.
Chitosan: the average of C1-2, C3-C2.
Mannan[1,2,3]: the average of C1-C2/3/4/5.
Xylose: the average of C1-C3, C3-2/5, C4-5.

Table S4. The molar composition of mobile polysaccharides. The numbers are estimated using integrals (volume) of cross peaks in 2D ^{13}C-^{13}C refocused DP-J INADEQUATE spectra. The average integrals of cross-peaks of each polysaccharide are shown. Error bars are standard errors of the peak integrals.

	Polysaccharide			
Strains	β-1,6-glucan	β-1,3-glucan	α-1,3-glucan (c)	Mannan
C. neoformans H99	71±11	21±9	4±1.0	4±0.9
C. neoformans pka1	77±13	17±5	3±0.5	3±0.7
C. neoformans JEC20	56±11	15±6	7±2	22±6
C. neoformans Cap70	68±13	19±8	4±1.0	9±1.6

The area of the following well-resolved cross peaks refocused DP-J INADEQUATE spectra are used:
β-1,6: the average of C1, C2, C3, C4, C5, and C6.
β-1,3; the average of C1, C2, C3, C4, C5, and C6.
α-1,3-glucan: the average of C1, C2, and C3.
Mannan[1,2]: the average of C3, C4, C5 and C6.

Table S5. Water-edited intensities of polysaccharides. Intensity ratios are obtained by comparing the peak intensities in water-edited and control spectra. The average values for each molecule in each sample are highlighted. Error bars are s.d. propagated from NMR signal-to-noise ratios.

Polysaccharide	Cross-peak	*C. neoformans* (Serotype A)		*C. neoformans* (Serotype D)	
		H99	*pka1*	JEC20	Cap70
α-1,3-glucan (A^a)	A1-1	0.11±0.06	0.31±0.03	0.33±0.04	0.37±0.02
	A1-3	0.58±0.08	0.15±0.08	0.39±0.01	0.31±0.06
	A1-2/5	0.29±0.02	0.24±0.04	0.30±0.05	0.42±0.03
	A1-A4	0.31±0.03	0.28±0.06	0.02±0.01	0.48±0.05
	A3-A1	0.34±0.08	0.19±0.05	0.33±0.02	0.32±0.07
	A3-A3	0.33±0.05	0.35±0.06	0.34±0.08	0.38±0.05
	A3-2/5	0.49±0.06	0.21±0.06	0.29±0.07	0.32±0.04
	A3-4	0.31±0.06	0.13±0.06	0.10±0.02	0.22±0.03
	A2/5-1	0.54±0.04	0.33±0.01	0.25±0.04	0.29±0.03
	A2/5-3	0.54±0.07	0.31±0.01	0.29±0.08	0.29±0.04
	A2/5-2/5	0.50±0.01	0.44±0.02	0.33±0.01	0.39±0.01
	A2/5-4	0.52±0.09	0.24±0.01	0.10±0.01	0.45±0.02
	A4-1	0.50±0.02	0.26±0.01	0.25±0.02	0.44±0.09
	A4-3	0.37±0.01	0.24±0.01	0.12±0.09	0.36±0.08
	A4-2/5	0.53±0.09	0.16±0.03	0.37±0.09	0.63±0.04
	A4-4	0.52±0.03	0.52±0.01	0.27±0.04	0.29±0.01
	Average	0.42	0.27	0.26	0.37
α-1,3-glucan (A^b)	A1-1	0.35±0.02	0.99±0.01	0.34±0.04	0.38±0.03
	A1-3	0.68±0.01	0.11±0.06	0.41±0.01	0.39±0.07
	A1-4	0.88±0.06	0.55±0.01	0.29±0.01	0.35±0.04
	A3-A1	0.14±0.03	0.23±0.01	0.31±0.01	0.32±0.07
	A3-3	0.40±0.06	0.37±0.06	0.34±0.08	0.38±0.05
	A3-4	0.13±0.01	0.48±0.02	0.24±0.06	0.28±0.04
	A4-1	0.39±0.04	0.31±0.06	0.21±0.01	0.43±0.05
	A4-3	0.40±0.06	0.24±0.07	0.29±0.02	0.39±0.05
	A4-4	0.49±0.01	0.42±0.01	0.32±0.08	0.43±0.01
	Average	0.43	0.41	0.31	0.37
α-1,3-glucan (A^c)	A1-1	0.61±0.03	0.23±0.01	0.37±0.04	0.53±0.04
	A1-3	0.94±0.08	0.57±0.07	0.21±0.02	0.61±0.07
	A1-4	0.02±0.09	0.01±0.01	0.69±0.08	0.99±0.08
	A3-A1	0.87±0.03	0.17±0.01	0.53±0.04	0.68±0.06
	A3-3	0.53±0.01	0.58±0.03	0.14±0.02	0.42±0.05
	A3-4	0.99±0.04	0.49±0.02	0.36±0.01	0.83±0.01
	A4-1	0.43±0.01	0.34±0.01	0.37±0.09	0.34±0.03
	A4-3	0.33±0.02	0.23±0.01	0.97±0.01	0.46±0.03
	A4-4	0.52±0.02	0.33±0.02	0.36±0.08	0.44±0.06
	Average	0.58	0.33	0.45	0.59
β-1,6-glucan (H)	H3-3	0.87±0.02	0.50±0.04	0.44±0.01	0.47±0.01
	H3-5	0.97±0.02	0.63±0.03	0.44±0.01	0.51±0.02
	H3-2	0.98±0.06	0.30±0.02	0.66±0.03	0.46±0.04
	H5-3	0.47±0.01	0.65±0.01	0.36±0.09	0.33±0.01
	H5-2	0.62±0.03	0.55±0.01	0.51±0.01	0.50±0.02
	H5-5	0.64±0.03	0.74±0.02	0.78±0.04	0.50±0.03
	H2-3	0.82±0.09	0.65±0.04	0.92±0.01	0.57±0.08

	H2-5	0.98±0.05	0.48±0.02	0.94±0.05	0.89±0.05
	H2-2	0.61±0.01	0.52±0.01	0.56±0.01	0.56±0.01
	Average	0.77	0.56	0.62	0.53
Chitin (Ch)	Ch1-1	0.57±0.04	0.10±0.01	0.41±0.01	0.27±0.02
	Ch1-3	0.93±0.06	0.12±0.02	0.54±0.03	0.68±0.02
	Ch1-2	0.88±0.04	0.41±0.02	0.49±0.02	0.59±0.03
	Ch3-1	0.79±0.02	0.54±0.03	0.45±0.02	0.72±0.01
	Ch3-3	0.85±0.02	0.34±0.02	0.36±0.02	0.64±0.02
	Ch3-2	0.64±0.04	0.53±0.08	0.91±0.02	0.62±0.05
	Ch2-1	0.93±0.01	0.71±0.02	0.02±0.04	0.99±0.01
	Ch2-3	0.64±0.07	0.53±0.03	0.68±0.02	0.91±0.06
	Ch2-2	0.51±0.02	0.58±0.01	0.59±0.07	0.56±0.01
	Average	0.75	0.43	0.49	0.67
Mannan (Mn[1,2,3])	Mn1-1	0.32±0.05	-	0.35±0.05	-
	Mn1-3	0.49±0.08	-	0.16±0.03	-
	Mn1-5	0.65±0.06	-	0.52±0.09	-
	Mn3-1	0.42±0.04	-	0.27±0.03	-
	Mn3-3	0.56±0.02	-	0.3±0.04	-
	Mn3-5	0.65±0.05	-	0.19±0.01	-
	Mn5-1	0.15±0.01	-	0.29±0.01	-
	Mn5-3	0.22±0.01	-	0.99±0.09	-
	Mn5-5	0.01±0.01	-	0.56±0.04	-
	Average	0.38		0.41	

Table S6. ^{1}H-$T_{1\rho}$ and ^{13}C-T_1 relaxation times of polysaccharides in cell walls. Data is shown for the *C. neoformans* samples. The average values for each molecule in each sample are highlighted in bold. The data were measured using 1D ^{13}C relaxation experiments. The data are fit using single exponential equations: $I(t) = e^{-t/T_1}$. Error bars are standard deviations of the fit parameters.

Polysaccharide	Chemical shift	H99		*pka1*		JEC20		Cap70	
		^{1}H-$T_{1\rho}$ (ms)	^{13}C-T_1 (s)	^{1}H-$T_{1\rho}$ (ms)	^{13}C-T_1 (s)	^{1}H-$T_{1\rho}$ (ms)	^{13}C-T_1 (s)	^{1}H-$T_{1\rho}$ (ms)	^{13}C-T_1 (s)
α-1,3-glucan (a)	101.5	11.4±1.2	3.6±0.2	13.6±1.0	3.4±0.1	18.6±1.3	3.8±0.2	14.0±1.1	4.8±0.4
	85.0	12.5±1.0	3.5±0.2	15.0±1.4	3.8±0.1	22.0±1.4	4.3±0.1	16.0±1.0	4.8±0.4
	71.9	8.5±0.9	2.8±0.3	9.8±0.9	2.5±0.2	12.4±1.0	3.0±0.2	10.8±0.9	3.3±0.3
	69.6	6.5±0.8	2.1±0.3	8.5±1.0	1.3±0.2	13.0±1.2	2.6±0.3	9.6±1.0	2.6±0.4
	Average	9.7	3.0	11.7	2.7	16.4	3.4	12.6	3.9
Chitin	104.6	4.9±0.6	1.3±0.1	6.9±1.1	2.5±0.5	4.7±0.8	1.5±0.3	7.9±1.2	2.2±0.4
	83.4	6.4±0.7	2.0±0.2	9.5±1.3	2.5±0.1	8.3±1.1	2.2±0.1	10.2±0.8	2.8±0.3
	55.9	3.8±0.5	0.9±0.1	7.6±0.8	1.4±0.09	8.0±0.9	1.0±0.1	7.3±1.0	1.4±0.2
	Average	5.1	1.4	8.0	2.1	7.0	1.6	8.5	2.1
β-1,6-glucan	103.9	4.2±0.4	1.1±0.1	5.9±0.7	0.7±0.08	6.3±0.6	1.4±0.2	5.0±0.6	1.0±0.2
	76.4	3.2±0.2	0.8±0.07	4.4±0.5	0.6±0.06	4.2±0.4	0.9±0.1	4.1±0.3	0.8±0.1
	75.6	3.4±0.3	0.9±0.09	4.7±0.5	0.8±0.08	4.3±0.5	1.4±0.1	4.5±0.4	1.1±0.1
	74.0	4.0±0.3	1.0±0.1	5.8±0.6	0.9±0.1	6.6±0.6	1.4±0.2	5.5±0.5	1.2±1.4
	70.6	7.0±0.8	2.0±0.2	7.9±0.8	1.3±0.2	13.0±0.9	2.4±0.2	9.2±0.9	2.4±0.3
	Average	4.4	1.2	5.8	0.9	6.8	1.5	5.6	1.3

Table S7. ^{1}H and ^{13}C chemical shifts of *C. neoformans* polysaccharides from ^{1}H-detected experiments. For each carbon site, the ^{13}C and ^{1}H chemical shifts are shown in the top and bottom rows, respectively. The referencing scale is TMS scale for ^{13}C, and DSS for ^{1}H. Underline: sites with ambiguity due to spectral overlap.

Carbohydrates	forms	C1/H1	C2/H2	C3/H3	C4/H4	C5/H5	C6/H6
Rigid molecules							
α-1,3-glucan (A)	a	101.6<br>5.5	71.2<br>4.2	85.5<br>3.5	71.2<br>4.2	71.2<br>4.2	61.2<br>4.4
	b	101.4<br>4.8	71.5<br>3.6	85.0<br>3.8	71.5<br>3.6	71.5<br>3.6	61.5<br>3.9
	e	101.2<br>6.1	71.5<br>3.2	85.0<br>4.4	71.5<br>3.2	71.5<br>3.2	61.2<br>3.4
β-1,6-glucan (H)		104<br>5.4-3.7	74.2<br>4.7-3.1	76.8<br>4.7-3.1	70.8<br>4.7-3.0	75.8<br>4.7-2.6	69.8<br>4.7-2.8

Table S8. ^{13}C chemical shifts of *C. neoformans* melanin from ^{1}H-detected experiments. For each aromatic carbon site, the ^{13}C chemical shifts are shown in ppm.

Melanin fragments	C4	C5	C6	C7	C8	C9
A	110.0-125.0	135.0	148.0	137.0	145.0	140.0-144.0
B	/	145.0	140.0-144.0	/	125.0-130.0	110.0-125.0
C	125.0-130.0	153.0	175.0-178.0	/	168.5	140.0-144.0
D	110.0-125.0	137.0	140.0-144.0	/	125.0-130.0	140.0-144.0

Table S9. ^{13}C chemical shifts of *C. neoformans* melanin from literature. For each group of aromatic carbons, the ^{13}C chemical shifts are shown in ppm.

Observed shifts (ppm)	Chemical grouping	Literature referencing
110-118	Aromatic -CH-	Vecchia *et al.* 2013[88]<br>Adhyaru *et al.* 2003[89]<br>Johnson *et al.* 2013[90]
125-130	-CH=CH-, indole or alkene	Vecchia *et al.* 2013[88]<br>Adhyaru *et al.* 2003[89]<br>Johnson *et al.* 2013[90]
144-156	Aromatic -CH=CHCO-, aromatic -C-	Vecchia *et al.* 2013[88]<br>Johnson *et al.* 2013[90]<br>Subhasish *et al.* 2014[91]
157-165	Aromatic -C-	Vecchia *et al.* 2013[88]<br>Johnson *et al.* 2013[90]
170-180	-COO-, -CONH	Vecchia *et al.* 2013[88]<br>Subhasish *et al.* 2014[91]

Table S10. ^{13}C Solid-state NMR experimental parameters for fungal cell wall characterization. T = sample temperature; B_0 = magnetic field; ν_{MAS} = MAS frequency; ns = number of scans; d_1 = recycle delay between scans; $t_{1,max}$ = maximum t_1 evolution time (for indirect dimension); $t_{1,inc}$ = increment for t_1 (for indirect dimension) evolution time; τ_{dw} = dwell time during direct FID acquisition; τ_{acq} = maximum acquisition time during direct FID detection; τ_{XY} = cross-polarization contact time during CP from channel X to channel Y; $\nu_{1H, dec}$ = dipolar decoupling field strength**.** Spin diffusion (SD).

Experiment	NMR Parameters															Samples
	T (K)	B_0 (T)	ν_{MAS} (kHz)	ns	d1 (s)	$t_{1,max}$ (ms)	$t_{1,inc}$ (μs)	τ_{dw} (μs)	τ_{acq} (ms)	τ_{HC} (ms)	τ_{HN} (ms)	τ_{NC} (ms)	τ_{SD} (ms)	τ_{mix} (ms)	ν_{1H} dec (kHz)	
Identification and quantification of polysaccharides																H99, JEC20, *pka1*, Cap70
1D ^{13}C CP	298	18.8	15	2048	2			5	18	1					83	
1D ^{13}C DP	298	18.8	15	512	2 or 35			5	18						83	
1D ^{13}C refocused INEPT	298	18.8	15	1024	3.5			5	16					1.7 τ_J	83	
2D ^{13}C-^{13}C with CORD mixing	298	18.8	15	32	2	7.5	25	5	14	0.5				53 τ_{CORD}	83	
2D ^{13}C-^{13}C refocused CP J-INADEQUATE	298	18.8	15	16	2	7.5	22	5	14						83	
2D ^{13}C-^{13}C refocused DP J-INADEQUATE	298	18.8	15	16	2	7.5	22	5	14						83	
Estimation of site-specific hydration of polysaccharides																
2D ^{13}C-^{13}C water-edited	280	9.4	15	64	2	5.5	50	8	16	1			0, 4	50 τ_{PDSD}	71	
Dynamics of polysaccharides																
1D ^{13}C-T_1	298	9.4	15	512	2			8	16	1					71	
1D ^{1}H-$T_{1\rho}$	298	9.4	15	512	2			8	16	1					71	
1D gated CP	298	14.1	14	1024	2			10	16	5					83	H99

Table S11. Parameters used for proton detection experiments. The CP based proton detection experiments were performed on 600 MHz (14.1 T) spectrometer with the MAS frequency of 60 kHz.

Expt.	Temp. (K)	CP (μs)		D1	NS	td2	td1	td3	aq2 (ms)	aq1 (ms)	aq3 (ms)	Water suppression	TOCSY(WALTZ-16) (ms)	RFDR Mixing (ms)	Sample
		t_{cp1}	t_{cp2}												
2D hCH	304	1000 (HC-CP)	100 (CH-CP)	3	32	1764 (^{1}H)	320 (^{13}C)	-	14.9	5.3	-	MISSISSIPI (total duration) 100 ms (rf 15.2 kHz)	-	-	Melanized *C. neoformans* H99
2D hChH (RFDR)	304	1000 (HC-CP)	500 (CH-CP)	2	512	1600 (^{1}H)	192 (^{13}C)	-	13.6	2.39	-		-	0.8	Melanized *C. neoformans* H99
3D hCCH TOCSY (WALTZ-16)	304	1000	100	2	8	1764	128	128	14.9	2.13	2.13	MISSISSIPI (total duration) 100 ms (rf 15.2 kHz	15 ms (rf 21.4 kHz)	-	Melanized *C. neoformans* H99
